# A rapid and efficient synthetic route to terminal arylacetylenes by tetrabutylammonium hydroxide- and methanol-catalyzed cleavage of 4-aryl-2-methyl-3-butyn-2-ols

**DOI:** 10.3762/bjoc.7.55

**Published:** 2011-04-13

**Authors:** Jie Li, Pengcheng Huang

**Affiliations:** 1 Department of Polymer Materials and Composites, School of Materials Science and Engineering, Beihang University, Beijing 100191, China

**Keywords:** 4-aryl-2-methyl-3-butyn-2-ol, deprotection reaction, 2-methyl-3-butyn-2-ol, terminal alkynes, tetrabutylammonium hydroxide

## Abstract

Tetrabutylammonium hydroxide with methanol as an additive was found to be a highly active catalyst for the cleavage of 4-aryl-2-methyl-3-butyn-2-ols. The reaction was performed at 55–75 °C and gave terminal arylacetylenes in good to excellent yields within several minutes. Compared with the usual reaction conditions (normally >110 °C, several hours), this novel catalyst system can dramatically decrease the reaction time under much milder conditions.

## Introduction

Terminal arylacetylenes are key precursors for the construction of conjugated oligo- or polyarylacetylenes, which have been widely used in the preparation of photoelectric devices such as organic light-emitting diodes (OLEDs) [1–4], field-effect transistors (OFETs) [5–6], and organic photovoltaic cells (OPVCs) [7–9]. The palladium-catalyzed Sonogashira cross-coupling of an aryl halide with a mono-protected acetylene and the subsequent removal of the protecting group is an important synthetic approach to access terminal arylacetylenes [10–13]. The commonly used mono-protected acetylenes are trialkylsilylacetylenes such as trimethylsilylacetylene (TMSA), triisopropylsilylacetylene (TIPSA) and [(3-cyanopropyl)dimethylsilyl]acetylene (CPDMSA), and 2-methyl-3-butyn-2-ol (MEBYNOL). The trialkylsilyl groups can be easily removed by treatment with oxygen-based nucleophiles or fluoride at ambient temperature [10–12]. However, trialkylsilylacetylenes are rather expensive that their use is limited to small-scale synthesis [13]. On the other hand, MEBYNOL ($ 0.3/g) is much cheaper than trialkylsilylacetylenes (TMSA $ 14.0/g, TIPSA $ 20.6/g, from Alfa Aesar). MEBYNOL couples with aryl halides in nearly quantitative yield and the coupling products 4-aryl-2-methyl-3-butyn-2-ols can be easily purified by chromatography because of the very different chromatographic polarities between the products and the aryl halides [13–22]. Nevertheless, the existing methods for the deprotection reaction (cleavage of 4-aryl-2-methyl-3-butyn-2-ols by removal of 2-hydroxypropyl group) to produce arylacetylenes often require harsh conditions [13–22], such as strong base (NaH, NaOH, KOH or *t*-BuOK), high temperature (in refluxing toluene or butanol) and a long reaction time. Such forcing conditions frequently lead to undesired side reactions and limit the application of MEBYNOL in the preparation of terminal arylacetylenes.

To solve the problem, herein we report a novel efficient catalyst system consisting of tetrabutylammonium hydroxide (Bu_4_NOH) and methanol for the removal of the 2-hydroxypropyl group from 4-aryl-2-methyl-3-butyn-2-ols under mild conditions. This deprotection reaction is remarkably facile (5–30 min, 55–75 °C) in comparison to the existing methods (usually several hours, >110 °C) [13–22] and gives the terminal arylacetylene products in good to excellent yields (up to 98%).

## Results and Discussion

Initially, we chose 4-(4-(phenylethynyl)phenyl)-2-methyl-3-butyn-2-ol (**1a**) as the model compound and carried out the deprotection reaction in toluene at 75 °C in the presence of inorganic bases and Bu_4_NOH/CH_3_OH, respectively (Table 1). It was found that by using inorganic bases, such as NaH, NaOH, NaOAc, KOH and K_2_CO_3_, no deprotected product **2a** was detected by TLC even after 24 h (Table 1, entries 1–5). Terminal arylacetylene **2a** was obtained in good yield only by NaOH with the addition of water and Bu_4_NI (the phase transfer catalyst), but this reaction required 23 h (Table 1, entry 6). Using Bu_4_NOH as the catalyst with CH_3_OH as an additive, **2a** was obtained in yields as high as 98% after only 5 min (Table 1, entry 7).

**Table 1:** Removal of 2-hydroxypropyl group in the presence of different catalysts^a^.

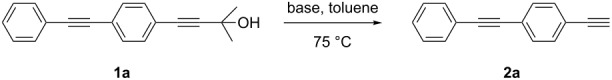			

Entry	Base (equiv)	Time (h)	Yield (%)^b^

1	KOH (5)	24	0
2	K_2_CO_3_ (5)	24	0
3	NaOAc (5)	24	0
4	NaH (5)	24	0
5	NaOH (5)	24	0
6	5 M aqueous NaOH (75) with Bu_4_NI (0.1)	23	89
7	Bu_4_NOH (0.1) with CH_3_OH (1.2)	0.08	98

^a^Reaction conditions: **1a** (2 mmol), base in toluene (100 mL) at 75 °C, under N_2_. ^b^Isolated yield.

The influence of the loading of Bu_4_NOH and CH_3_OH, as well as the effect of temperature on the yield of **2a** were then investigated (Table 2). The yield decreased from 98% to 29% when the Bu_4_NOH loading relative to **1a** decreased from 10 mol % to 2.5 mol % (Table 2, entries 1, 2 and 5), suggesting that 10 mol % of Bu_4_NOH was required. The optimum quantity of CH_3_OH was 1.2 equiv with respect to the amount of **1a**. The yield increased with increasing CH_3_OH loading when the mole ratio CH_3_OH/**1a** was lower than 1.2 (Table 2, entries 2–4), but it decreased with increasing CH_3_OH loading when the mole ratio CH_3_OH/**1a** was higher than 1.2 (Table 2, entries 5–7). Using 10 mol % of Bu_4_NOH and 1.2 equiv of CH_3_OH, **2a** was obtained in good to excellent yields in a relatively wide temperature range of 55–85 °C (Table 2, entries 5, 8–13). It reached the highest yield of 98% at 75 °C within 5 min. The yield was reduced significantly when the temperature was lower than 55 °C. Compared to the conventional method (>110 °C), this lower reaction temperature of 55–75 °C is especially meaningful for the 4-aryl-2-methyl-3-butyn-2-ols containing temperature-sensitive groups.

**Table 2:** Effect of reaction conditions on the yield of **2a**.

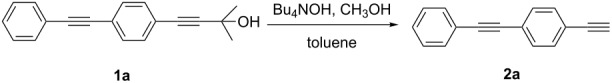					

Entry	Bu_4_NOH (mol %)	CH_3_OH (equiv)	Temp (°C)	Time (min)	Yield (%)^b^

1	2.5	1.2	75	45	29
2	5	1.2	75	30	80
3	5	1.0	75	30	70
4	5	0.6	75	30	68
5	10	1.2	75	5	98
6	10	1.9	75	5	92
7	10	3.2	75	5	80
8	10	1.2	85	5	92
9	10	1.2	65	5	95
10	10	1.2	55	30	87
11	10	1.2	45	30	28
12	10	1.2	35	30	15
13	10	1.2	25	600	0

^a^Reaction conditions: **1a** (2 mmol), Bu_4_NOH and CH_3_OH in toluene (100 mL), under N_2_. ^b^Isolated yield.

Thus, the optimal deprotection reaction required 10 mol % of Bu_4_NOH with 1.2 equiv of CH_3_OH in toluene at 75 °C. Then the deprotection reaction was investigated for different 4-aryl-2-methyl-3-butyn-2-ols. As shown in Table 3, this method was rapid, efficient and worked well with a broad range of 4-aryl-2-methyl-3-butyn-2-ols. Regarding the most studied 4-aryl-2-methyl-3-butyn-2-ols (Table 3, entries 1–4, 6, 7 and 9–11), the deprotection reaction was complete within 5 min at 75 °C, giving the terminal arylacetylenes in good to excellent yields. Products phenylacetylene (**2f**) was a liquid with a boiling point of 142 °C and 4-bromophenylacetylene (**2g**) was a solid that sublimed, consequently some of these compounds were lost during purification (Table 3, entries 6 and 7). Arylacetylenes **1e** and **1h** bearing electron-donating groups showed low reactivity [22], and the reactions required heating at 75 °C for 15 min then at 90 °C for 15 min (Table 3, entries 5 and 8). However, the reaction conditions were still milder than those of existing methods (>3 h) and satisfactory yields were achieved. It is worth mentioning that for the 4-aryl-2-methyl-3-butyn-2-ols containing two or more butynols, complete deprotection was achieved in only 5–30 min with good yields (Table 3, entries 8–11) using our method, whilst by using the conventional method deprotection was incomplete or the yields were low, or longer reaction times were required [22–25]. For example, multifunctional arylacetylenes **2i** and **2k** were prepared in 85% and 83% yields in 5 min (Table 3, entries 10 and 11), whereas they were obtained in only 38% and 11% yields in 8 h, respectively, when sodium was used in refluxing toluene (Supporting Information File 1). Thus, our novel catalyst system may find applications for the preparation of arylacetylene dendritic macromolecules in high yield.

**Table 3:** Synthesis of different terminal arylacetylenes by removal of 2-hydroxypropyl groups under optimal conditions^a^.

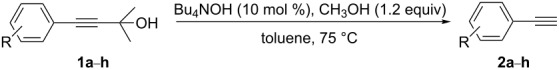				

Entry	4-Aryl-2-methyl-3-butyn-2-ol **1**	Terminal arylacetylene **2**	Time (min)	Yield (%)^b^

1	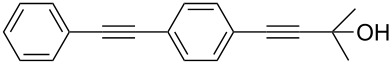 **1a**	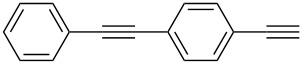 **2a**	5	98
2	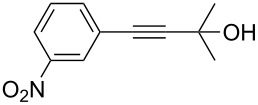 **1b**	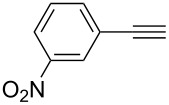 **2b**	5	88
3	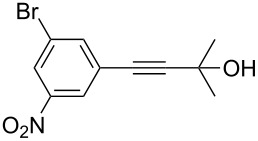 **1c**	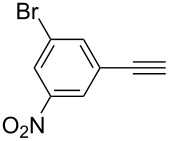 **2c**	5	85
4	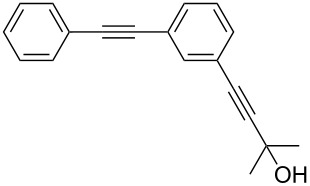 **1d**	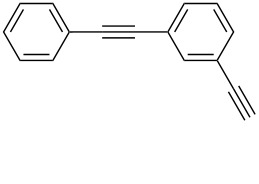 **2d**	5	89
5	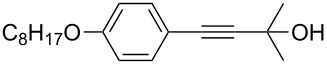 **1e**	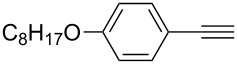 **2e**	30^c^	95
6	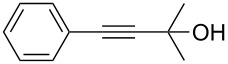 **1f**	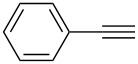 **2f**	5	88
7	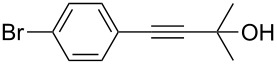 **1g**	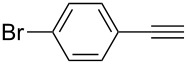 **2g**	5	74
8	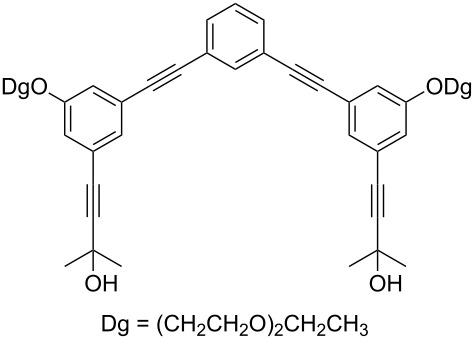 **1h**	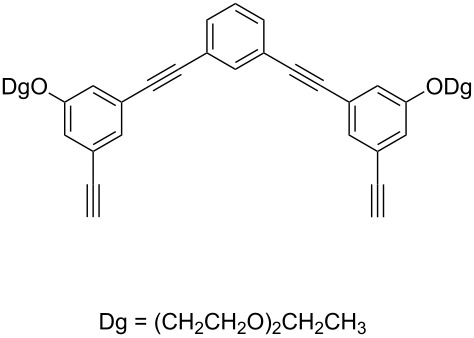 **2h**	30^c^	74
9	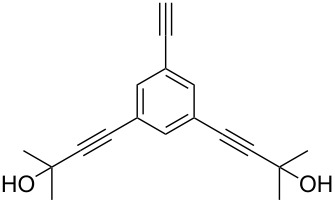 **1i**	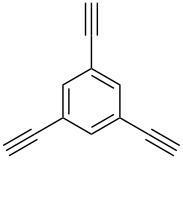 **2i**	5	91
10	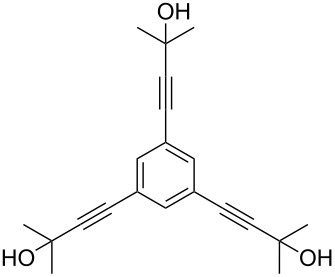 **1j**	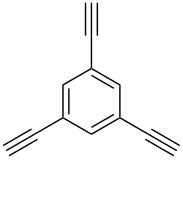 **2i**	5	85
11	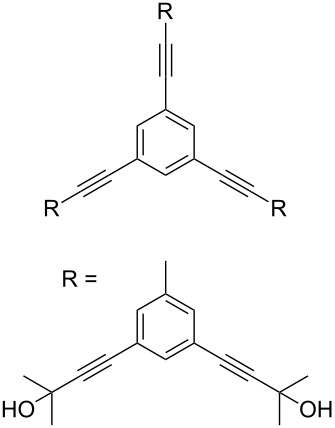 **1k**	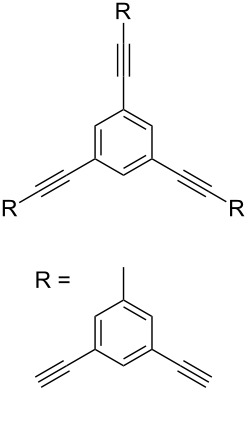 **2k**	5	83

^a^Reaction conditions: 4-aryl-2-methyl-3-butyn-2-ol (2 mmol), Bu_4_NOH (0.1 mol per 1 mol 2-hydroxypropyl group), CH_3_OH (1.2 mol per 1 mol 2-hydroxypropyl group) and toluene (100 mL) under a N_2_ atmosphere. ^b^Isolated yields. ^c^The reaction was carried out at 75 °C for 15 min, then at 90 °C for 15 min.

## Conclusion

In summary, a rapid, simple, and efficient method has been developed for the preparation of terminal arylacetylenes by the removal of the 2-hydroxypropyl group from 4-aryl-2-methyl-3-butyn-2-ols. By using 10 mol % Bu_4_NOH as the catalyst with the addition of 1.2 equiv of CH_3_OH at 55–75 °C complete deprotection was achieved within several minutes with good to excellent yields (up to 98%). This method has good functional group tolerance and shows significant promise for the preparation of arylacetylenes with multiple terminal ethynyl groups such as dendritic macromolecules, which will broaden the application of the low-cost reagent 2-methyl-3-butyn-2-ol in the preparation of arylacetylenes.

## Experimental

**General procedure for the deprotection reaction using Bu_4_NOH as base with CH_3_OH in toluene:** Under a nitrogen atmosphere, 4-aryl-2-methyl-3-butyn-2-ol (2 mmol) was dissolved into anhydrous toluene (100 mL) and the solution was heated to 75 °C, then Bu_4_NOH, 40 wt % solution in CH_3_OH (Bu_4_NOH: 0.1 mol per 1 mol 2-hydroxypropyl group) was added. The mixture was stirred at 75 °C for the indicated time. After cooling down to room temperature, the mixture was washed successively with 5% HCl and brine, dried over MgSO_4_, and concentrated in vacuo. The crude product was then purified by column chromatography to afford the product.

## Supporting Information

File 1 General experimental methods, analytical data, ^1^H and ^13^C NMR spectra of compounds **1a**–**k** and **2a**–**k**.
