# Supplementary material for: A rapid and efficient synthetic route to terminal arylacetylenes by tetrabutylammonium hydroxide- and methanol-catalyzed cleavage of 4-aryl-2-methyl-3-butyn-2-ols
Source: Beilstein J Org Chem. 2011 Apr 13;7:426–31. doi: 10.3762/bjoc.7.55 (PMC3079113; doi:10.3762/bjoc.7.55)
Supplement: File 1 — General experimental methods, analytical data, 1H and 13C NMR spectra of compounds 1a–k and 2a–k. [file Beilstein_J_Org_Chem-07-426-s001.pdf]

Supporting Information

for

A rapid and efficient synthetic route to terminal arylacetylenes by tetrabutylammonium hydroxide- and methanol-catalyzed cleavage of 4-aryl-2-methyl-3-butyn-2-ols

Jie Li and Pengcheng Huang*

Address: Department of Polymer Materials and Composites, School of Materials Science and Engineering, Beihang University, Beijing 100191, China

Email: Jie Li - lijie1159@mse.buaa.edu.cn; Pengcheng Huang* - huangpc@buaa.edu.cn

* Corresponding author

General experimental methods, analytical data, ^{1}H and ^{13}C NMR spectra of compounds 1a–k and 2a–k.

Experimental procedures and characterization data for the products:

General methods

Unless otherwise stated, all starting materials were obtained from commercial suppliers and were used without further purification. 4-Aryl-2-methyl-3-butyn-2-ols were prepared according to the general procedure. Toluene and triethylamine were distilled from CaH_2 prior to use. All reactions were performed under an atmosphere of nitrogen. Melting points were taken on a hot-plate microscope apparatus and are uncorrected. ^{1}H (300 MHz) and ^{13}C (75 MHz) NMR spectra were acquired on a Bruker AV 300 NMR spectrometer.

General procedure for the synthesis of 4-aryl-2-methyl-3-butyn-2-ols

A mixture of aryl bromide (5.0 mmol), 2-methyl-3-butyn-2-ol (1.0 mol per 1 mol bromine atom), copper(I) iodide (38.2 mg, 0.2 mmol), palladium(II) dichlorobistriphenylphosphine (70.2 mg, 0.1 mmol) and triphenylphosphine (104.9 mg, 0.4 mmol) in triethylamine (25 mL) was placed in a Schlenk flask. The mixture was heated at 65 °C for 6 h. After cooling to room temperature, the solvent was removed under reduced pressure. The residue was dissolved in dichloromethane and the organic phase was washed successively with 5% HCl and brine, dried over $MgSO_4$, and then evaporated. The crude product was purified by column chromatography with petroleum ether–ethyl acetate mixtures as eluent.

General procedure for the synthesis of 2i and 2k using sodium in refluxing toluene

4-Aryl-2-methyl-3-butyn-2-ol (2 mmol) and Na (2 mol per 1 mol 2-hydroxypropyl group) were dissolved in toluene (100 mL). The mixture was stirred at 120 °C for 8 h. After cooling to room temperature, the solution was washed successively with 5% HCl and brine, dried over $MgSO_4$, and concentrated in vacuo. The crude product was then purified by column chromatography to afford the product.

Characterization data of 1a–k and 2a–k

4-(4-(Phenylethynyl)phenyl)-2-methyl-3-butyn-2-ol (1a): eluent, petroleum ether–ethyl acetate = 10/1; yield: 81%; white solid; mp 135.2–135.7 °C; ^{1}H NMR ($CDCl_3$) δ 7.46–7.53 (m, 4H), 7.34–7.40 (m, 5H), 1.78 (s, 1H), 1.63 (s, 6H); ^{13}C NMR ($CDCl_3$) δ 31.45, 65.66, 81.87, 88.98, 91.12, 95.51, 122.58, 123.04, 123.19, 128.38, 128.46, 131.44, 131.59, 131.63.

4-(3-Nitrophenyl)-2-methyl-3-butyn-2-ol (1b): CAS NO.: 33432-52-9; eluent, petroleum ether–ethyl acetate = 5/1; yield: 90%; white solid; mp 51.7–52.2 °C; ^{1}H NMR ($CDCl_3$) δ 8.28 (t, *J* = 2 Hz, 1H), 8.17 (d, *J* = 8.4 Hz, 1H), 7.73 (d, *J* = 7.6 Hz, 1H), 7.50 (t, *J* = 8 Hz, 1H), 1.83 (s, 1H), 1.65 (s, 6H); ^{13}C NMR ($CDCl_3$) δ 31.29, 65.56, 79.84, 96.46, 122.95, 124.63, 126.47, 129.28, 137.29, 148.18.

4-(3-Bromo-5-nitrophenyl)-2-methyl-3-butyn-2-ol (1c): eluent, petroleum ether–ethyl acetate = 5/1; yield: 68%; white solid; mp 68.2–68.8 °C; ^{1}H NMR ($CDCl_3$) δ 8.28 (t, *J* = 1.8 Hz, 1H), 8.17 (t, *J* = 1.8 Hz, 1H), 7.85 (t, *J* = 1.8 Hz, 1H), 2.32 (s, 1H), 1.63 (s, 6H); ^{13}C NMR ($CDCl_3$) δ 31.20, 65.54, 78.65, 97.95, 122.59, 125.10, 126.14, 126.18, 139.93, 148.58.

4-(3-(Phenylethynyl)phenyl)-2-methyl-3-butyn-2-ol (1d): eluent, petroleum ether–ethyl acetate = 6/1; yield: 63%; white solid; mp 67–67.5 °C; ^{1}H NMR ($CDCl_3$) δ 7.62 (s, 1H), 7.46–7.53 (m, 3H), 7.30–7.39 (m, 5H), 1.72 (s, 1H), 1.64 (s, 6H); ^{13}C NMR ($CDCl_3$) δ 31.46, 65.62, 81.39, 88.48, 90.01, 94.39, 123.02, 123.11, 123.58, 128.39, 128.46, 131.31, 131.65, 134.71.

4-(4-Octyloxyphenyl)-2-methyl-3-butyn-2-ol (1e): CAS NO.: 125151-57-7; eluent, petroleum ether–ethyl acetate = 10/1; yield: 75%; white solid; mp 63.1–63.2 °C; ^{1}H MNR ($CDCl_3$) δ 7.33 (d, *J* = 9 Hz, 2H), 6.80 (d, *J* = 8.7 Hz, 2H), 3.93 (t, *J* = 6.9 Hz, 2H), 2.12 (s, 1H), 1.72–1.81 (m, 2H), 1.60 (s, 6H), 1.29–1.46 (m, br, 10H), 0.89 (t, *J* = 6.9 Hz, 3H); ^{13}C NMR ($CDCl_3$) δ 14.05, 22.63, 26.00, 29.18, 29.19, 29.32, 31.59, 31.79, 65.65, 68.08, 82.13, 92.32, 114.44, 114.59, 133.04, 159.20.

4-Phenyl-2-methyl-3-butyn-2-ol (1f): CAS NO.: 1719-19-3; eluent, petroleum ether–ethyl acetate = 10/1; yield: 82%; white solid; mp 52.4–53 °C; ^{1}H MNR ($CDCl_3$) δ 7.40–7.43 (m, 2H), 7.28–7.30 (m, 3H), 2.07 (s, 1H), 1.62

(s, 6H); ^{13}C NMR ($CDCl_3$) δ 31.50, 65.62, 82.25, 93.82, 122.78, 128.24, 131.65.

4-(4-Bromophenyl)-2-methyl-3-butyn-2-ol (1g): CAS NO.: 76347-62-1; eluent, petroleum ether–ethyl acetate = 6/1; yield: 62%; white solid; mp 59.5–61 °C; ^{1}H MNR ($CDCl_3$) δ 7.42 (d, *J* = 8.1 Hz, 2H), 7.26 (d, *J* = 8.4 Hz, 2H), 2.03 (s, 1H), 1.61 (s, 6H); ^{13}C NMR ($CDCl_3$) δ 31.40, 65.62, 81.16, 94.97, 121.74, 122.49, 131.52, 133.09.

1,3-Bis{3-(3-methyl-3-hydroxy-butynyl)-5-[2-(2-ethoxyethoxy)ethoxy] phenylethynyl}benzene (1h): golden yellow oil; ^{1}H NMR ($CDCl_3$) δ 7.64 (s, 1H), 7.44 (d, *J* = 7.5 Hz, 2H), 7.30 (t, *J* = 7.5 Hz, 1H), 7.18 (s, 2H), 7.00 (s, 2H), 6.93 (s, 2H), 4.08–4.13 (m, 4H), 3.84 (t, *J* = 4.6 Hz, 4H), 3.70 (t, *J* = 4.6 Hz, 4H), 3.59 (t, *J* = 4.6 Hz, 4H), 3.52 (q, *J* = 6.9 Hz, 4H), 2.02 (s, 2H), 1.59 (s, 12H), 1.17–1.22 (m, 6H); ^{13}C NMR ($CDCl_3$) δ 15.14, 31.43, 65.52, 66.70, 67.81, 69.59, 69.86, 70.97, 81.25, 88.77, 89.15, 94.32, 117.95, 118.21, 123.38, 124.08, 124.14, 127.65, 128.54, 131.50, 134.68, 158.46.

1-Ethynyl-3,5-bis[(2-hydroxyprop-2-yl)ethynyl]benzene (1i): white solid; mp 129.7–130.3 °C (lit. [1] 95–97 °C); ^{1}H MNR ($CDCl_3$) δ 7.42–7.44 (m, 3H), 3.06 (s, 1H), 1.82 (s, 2H), 1.56 (s, 12H); ^{13}C NMR ($CDCl_3$) δ 31.36, 65.54, 78.30, 80.43, 81.89, 95.10, 122.72, 123.44, 134.63, 134.82.

1,3,5-Tris[(2-hydroxyprop-2-yl)ethynyl]benzene (1j): eluent, petroleum ether–ethyl acetate = 1/1; yield: 89%; white solid; mp 169–170 °C (lit. [1] 170–

172 °C); ^{1}H MNR ($CDCl_3$) δ 7.40 (s, 3H), 1.59 (s, 21H); ^{13}C NMR ($CDCl_3$) δ 31.37, 65.54, 80.55, 94.93, 123.34, 134.23.

3-Cascade:benzene[3-1,3,5]:5-ethynyl-1,3-bis-[(2-hydroxyprop-2-yl)ethynyl]benzene (1k): white solid; mp >300 °C (decompose); ^{1}H MNR (DMSO-d_6) δ 7.83 (s, 3H), 7.54 (d, J = 1.5 Hz, 6H), 7.40 (s, 3H), 5.53 (s, 6H), 1.47 (s, 36H); ^{13}C NMR (DMSO-d_6) δ 31.39, 63.61, 78.48, 88.59, 89.09, 97.88, 122.91, 123.25, 123.91, 133.33, 134.04, 134.45.

1-Ethynyl-4-(phenylethynyl)benzene (2a): CAS NO.: 92866-00-7; eluent, petroleum ether; yield: 97.6%; white solid; mp 91.6–92.1 °C; ^{1}H MNR ($CDCl_3$) δ 7.48–7.54 (m, 6H), 7.35–7.36 (m, 3H), 3.17 (s, 1H); ^{13}C NMR ($CDCl_3$) δ 78.86, 83.30, 88.84, 91.40, 121.90, 122.99, 123.83, 128.40, 128.54, 131.48, 131.66, 132.08.

1-Ethynyl-3-nitrobenzene (2b): CAS NO.: 3034-94-4; eluent, petroleum ether; yield: 88.2%; yellow oil; ^{1}H MNR ($CDCl_3$) δ 8.27 (t, J = 1.8 Hz, 1H), 8.14 (d, J = 8.3 Hz, 1H), 7.72 (d, J = 7.7 Hz, 1H), 7.45 (t, J = 8 Hz, 1H), 3.15 (s, 1H); ^{13}C NMR ($CDCl_3$) δ 79.90, 81.10, 123.57, 123.95, 126.99, 129.39, 137.77, 148.20.

1-Ethynyl-3-bromo-5-nitrobenzene (2c): eluent, petroleum ether–ethyl acetate= 20/1; yield: 85.1%; yellow solid; mp 77.4–77.9 °C; ^{1}H MNR ($CDCl_3$) δ 8.35 (s, 1H), 8.26 (s, 1H), 7.92 (s, 1H), 3.28 (s, 1H); ^{13}C NMR ($CDCl_3$) δ 79.81, 81.37, 122.70, 125.43, 125.62, 126.86, 140.39, 148.61.

1-Ethynyl-3-(phenylethynyl)benzene (2d): eluent, petroleum ether; yield: 88.9%; colorless oil; ^{1}H MNR ($CDCl_3$) δ 7.68 (s, 1H), 7.45–7.54 (m, 4H), 7.30–7.37 (m, 4H), 3.11 (s, 1H); ^{13}C NMR ($CDCl_3$) δ 77.79, 82.82, 88.35, 90.25, 122.53, 122.99, 123.72, 128.41, 128.44, 128.51, 131.69, 131.80, 131.86, 135.13.

1-Ethynyl-4-octyloxybenzene (2e): CAS NO.: 79887-19-7; eluent, petroleum ether; yield: 95.3%; colorless oil; ^{1}H MNR ($CDCl_3$) δ 7.43 (d, J = 8.6 Hz, 2H), 6.84 (d, J = 8.6 Hz, 2H), 3.97 (t, J = 6.5 Hz, 2H), 3.01 (s, 1H), 1.76–1.83 (m, 2H), 1.30–1.47 (m, br, 10H), 0.90 (t, J = 6.2 Hz, 3H); ^{13}C NMR ($CDCl_3$) δ 14.07, 22.64, 26.01, 29.17, 29.22, 29.33, 31.80, 68.09, 75.63, 83.79, 113.94, 114.49, 133.56, 159.59.

Ethynylbenzene (2f): CAS NO.: 536-74-3; eluent, petroleum ether; yield: 88%; colorless oil; ^{1}H MNR ($CDCl_3$) δ 7.48–7.50 (m, 2H), 7.30–7.32 (m, 3H), 3.06 (s, 1H); ^{13}C NMR ($CDCl_3$) δ 77.48, 83.69, 122.18, 128.33, 128.81, 132.17.

4-Bromophenylacetylene (2g): CAS NO.: 766-96-1; eluent, petroleum ether; yield: 73.6%; white solid; mp 64.1–65 °C; ^{1}H MNR ($CDCl_3$) δ 7.46 (d, J = 8.4 Hz, 2H), 7.36 (d, J = 8.4 Hz, 2H), 3.13 (s, 1H); ^{13}C NMR ($CDCl_3$) δ 78.33, 82.60, 121.06, 123.25, 131.61, 133.56.

1,3-Bis{[3-ethynyl-5-[2-(2-ethoxyethoxy)ethoxy]phenyl]ethynyl}benzene (2h): eluent, petroleum ether–ethyl acetate= 4/1; yield: 74.4%; pale yellow

viscous oil; ^{1}H NMR ($CDCl_3$) δ 7.61 (s, 1H), 7.41 (d, *J* = 7.8 Hz, 2H), 7.26–7.19 (m, 3H), 7.00–6.96 (m, 4H), 4.08 (t, *J* = 4.5 Hz, 4H), 3.80 (t, *J* = 4.8 Hz, 4H), 3.65 (t, *J* = 4.8 Hz, 4H), 3.55 (t, *J* = 4.8 Hz, 4H), 3.47 (q, *J* = 6.9 Hz, 4H), 3.01 (s, 2H), 1.15 (t, *J* = 6.9 Hz, 6H); ^{13}C NMR ($CDCl_3$) δ 15.16, 66.71, 67.81, 69.58, 69.87, 70.98, 77.68, 82.67, 88.89, 89.02, 118.40, 118.75, 123.32, 123.42, 124.25, 128.03, 128.57, 131.58, 134.70, 158.48.

1,3,5-Triethynylbenzene (2i): CAS NO.: 7567-63-7; eluent, petroleum ether; white solid; mp 106–107.2 °C; ^{1}H MNR ($CDCl_3$) δ 7.57 (s, 3H), 3.10 (s, 3H); ^{13}C NMR ($CDCl_3$) δ 78.67, 81.63, 122.96, 135.65.

1,3,5-Tris[2-(3,5-diethynylphenyl)ethynyl]benzene (2k): eluent, THF. The product was further purified by precipitation of a THF solution into 20 volumes of methanol to give **2k** as a white fluffy solid. Yield: 82.6%; ^{1}H NMR (DMSO-d_6): δ 7.62-7.84 (m, 12H), 4.39(s, 6H); ^{13}C NMR (DMSO-d_6) δ 81.31, 82.68, 88.76, 88.85, 123.00, 123.10, 134.49, 134.60, 134.91.

References

1. Rodríguez, J. G.; Esquivias, J. *Tetrahedron Lett.* **2003,** *44*, 4831–4834. doi:10.1016/S0040-4039%2803%2901137-7

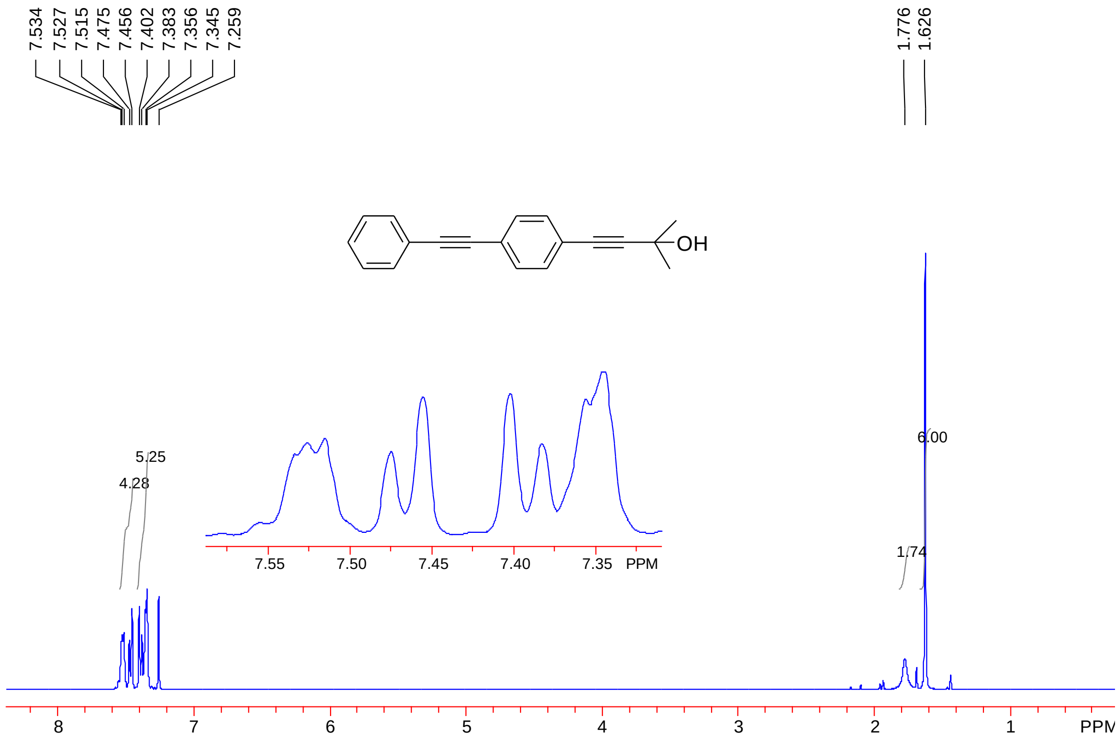

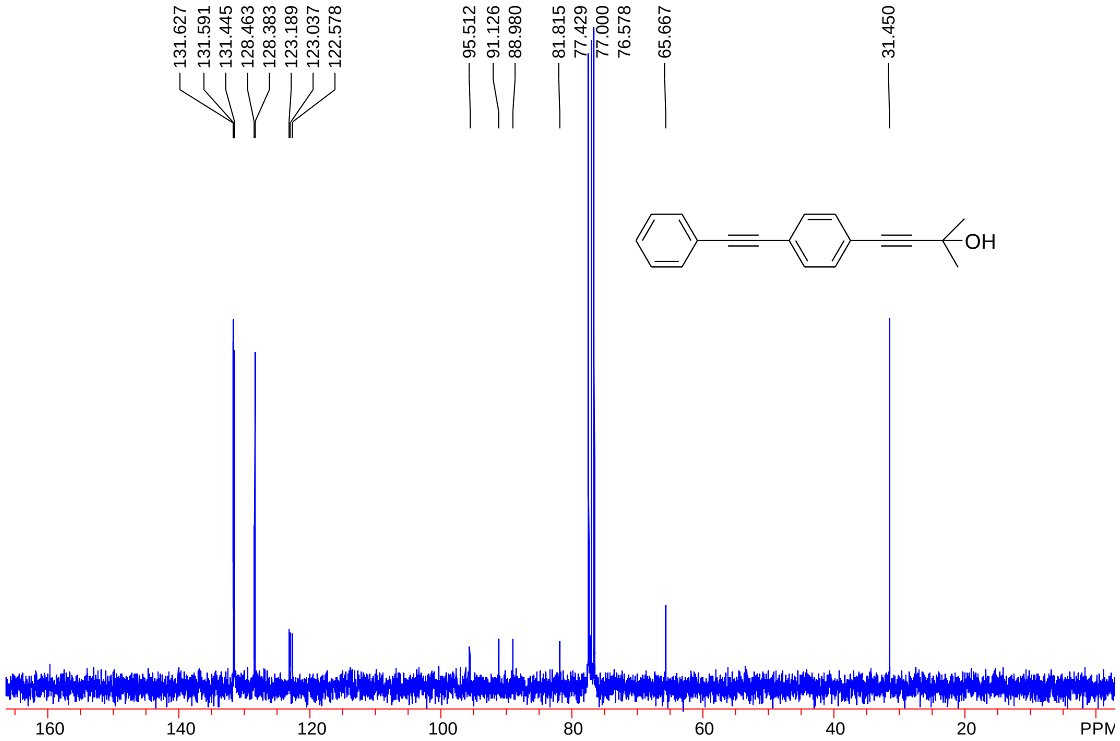

Figure S1: ^{1}H and ^{13}C NMR spectra of **1a**.

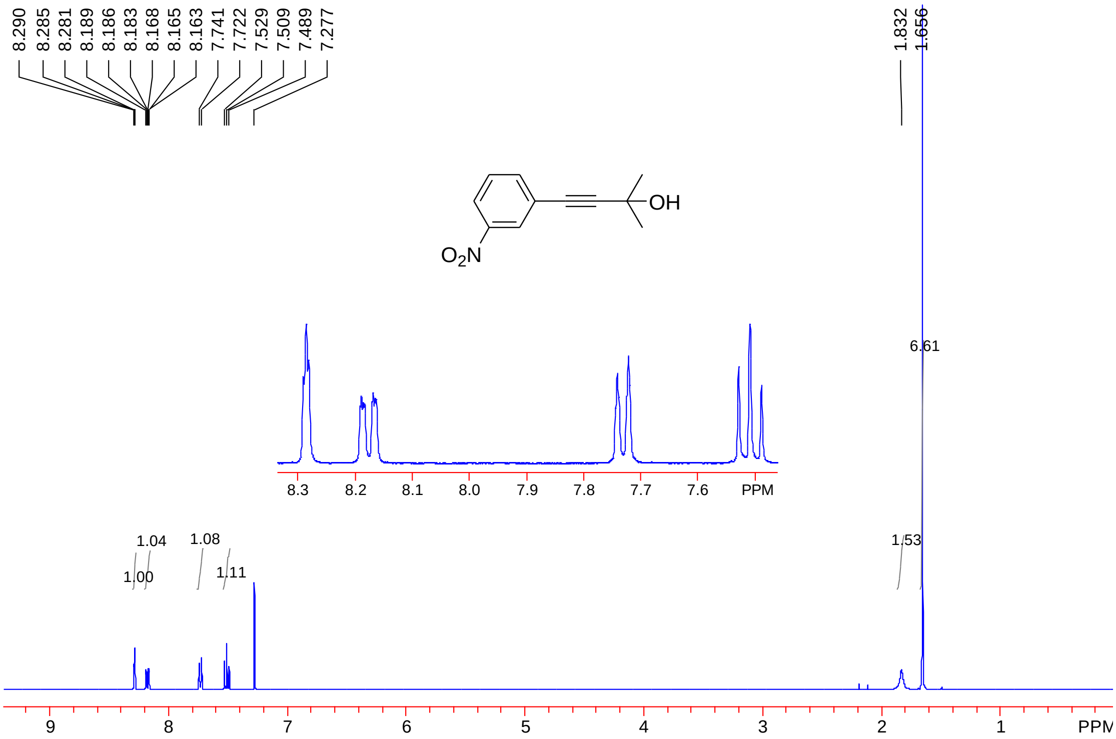

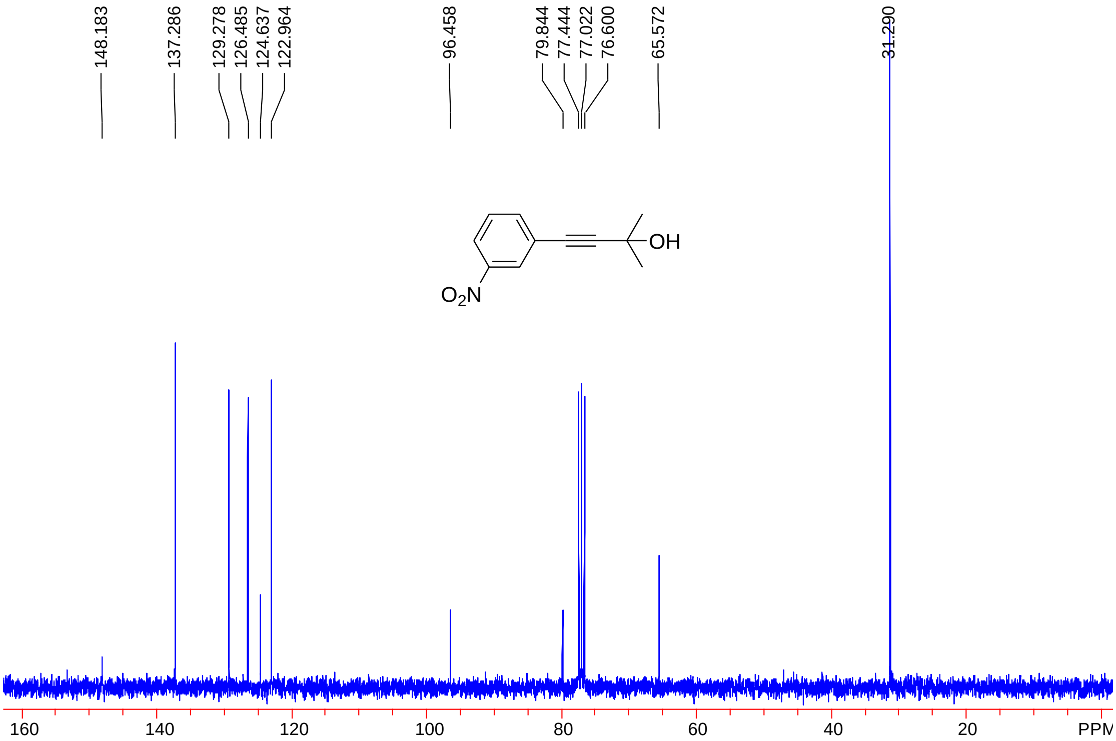

Figure S2: ^{1}H and ^{13}C NMR spectra of **1b**.

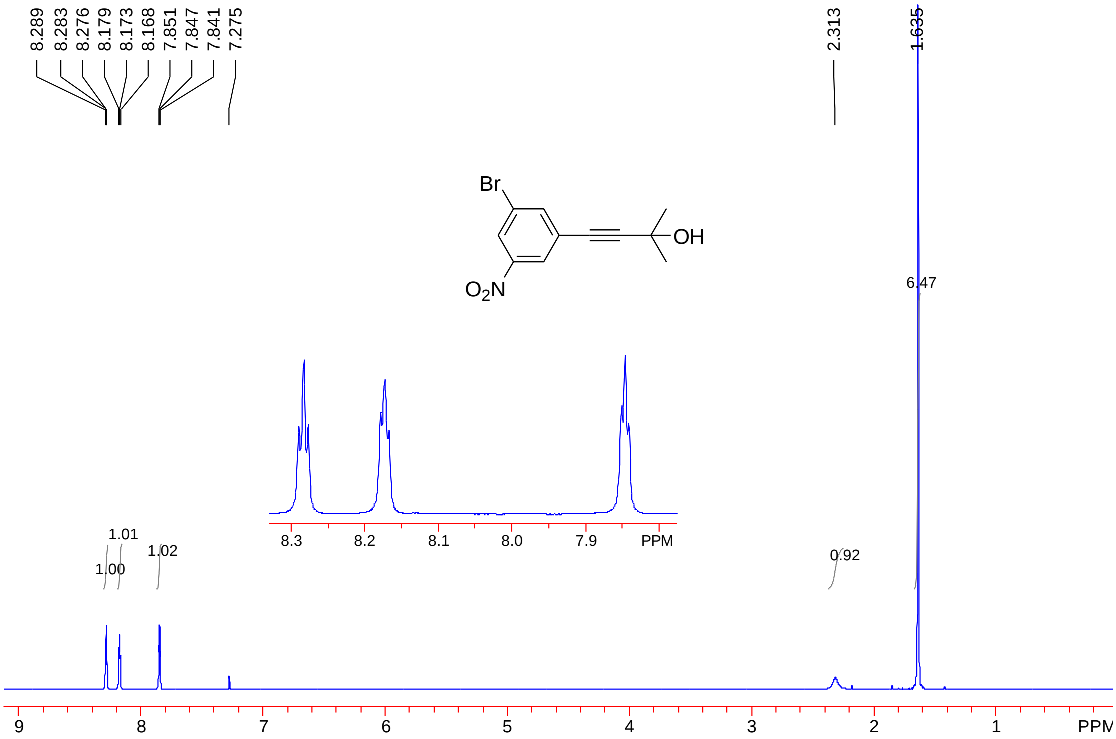

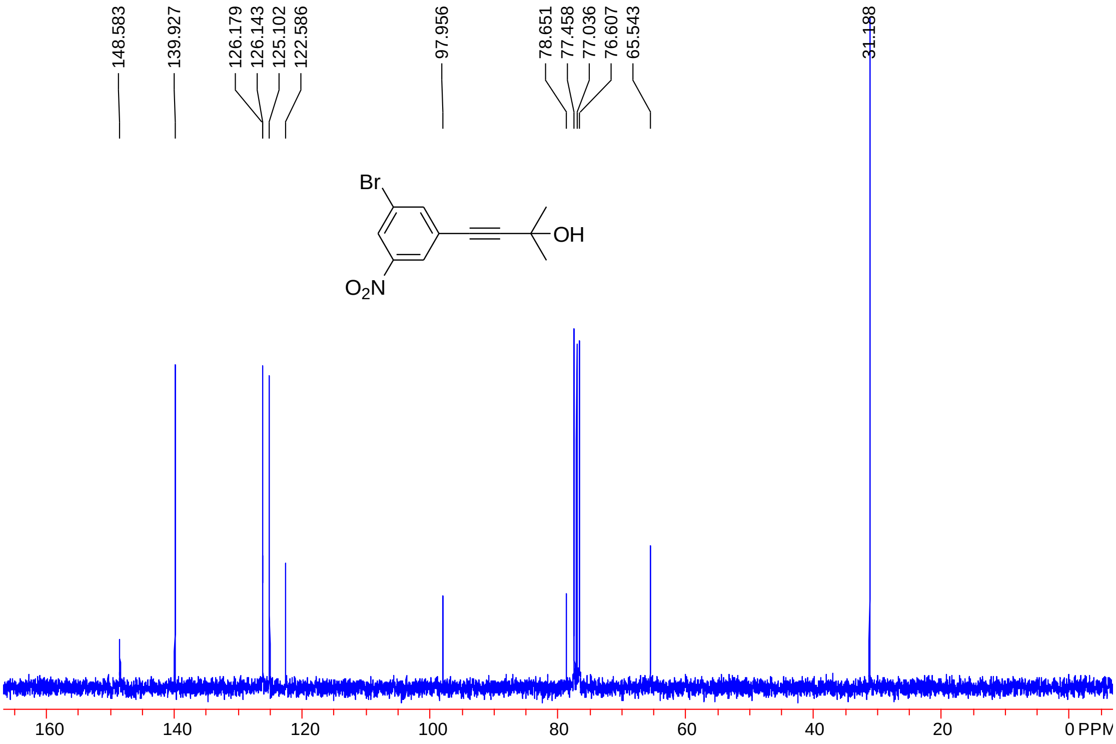

Figure S3: ^{1}H and ^{13}C NMR spectra of **1c**.

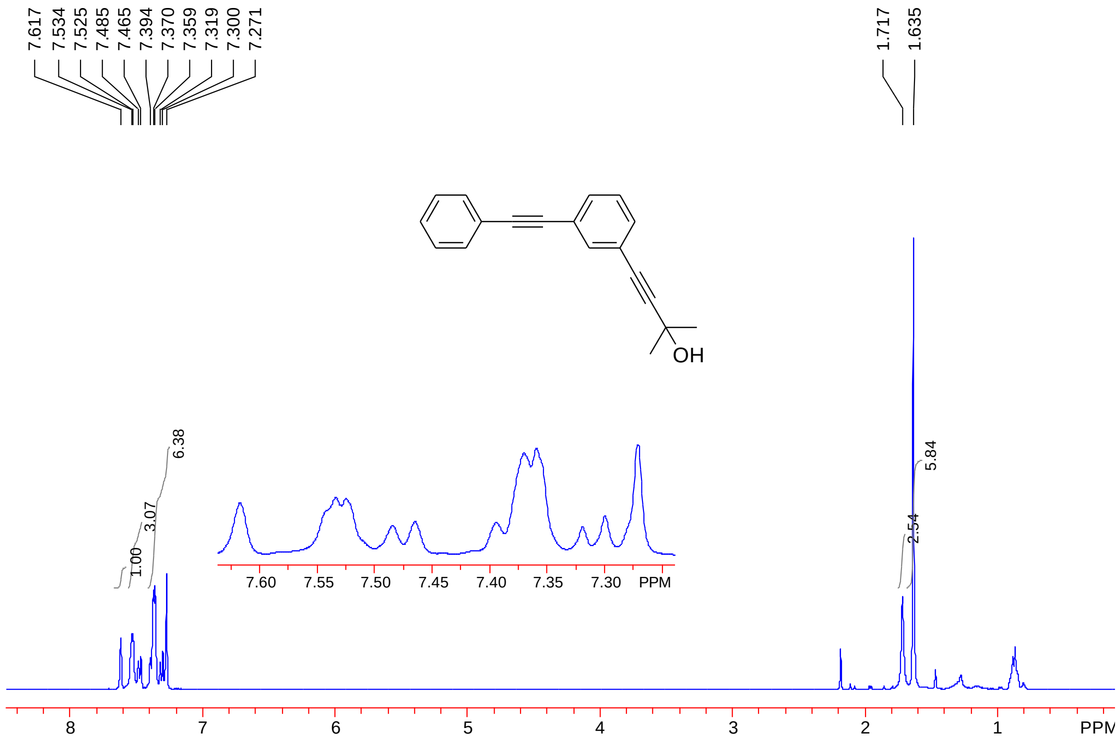

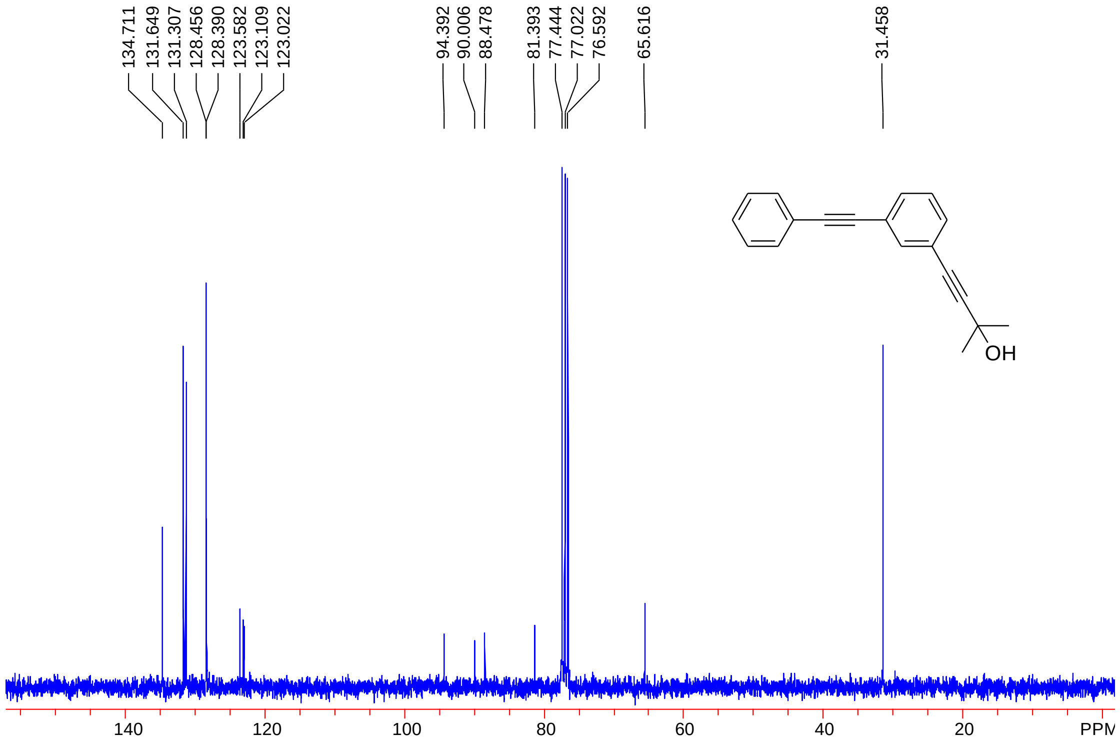

Figure S4: ^{1}H and ^{13}C NMR spectra of **1d**.

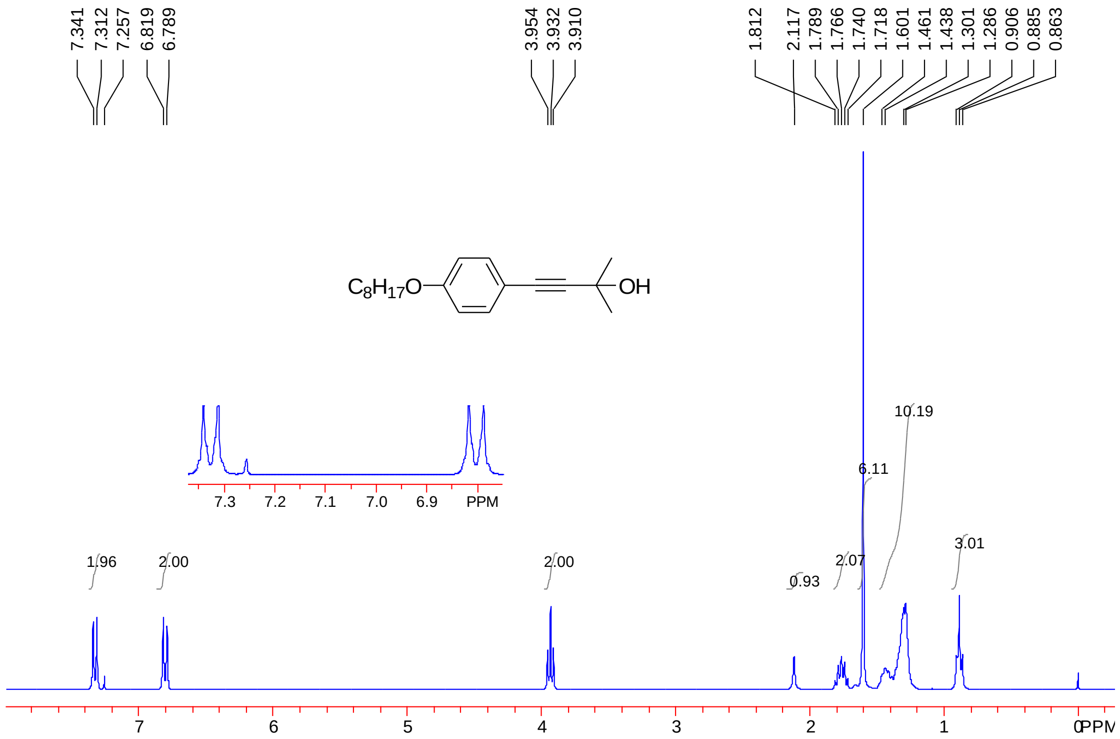

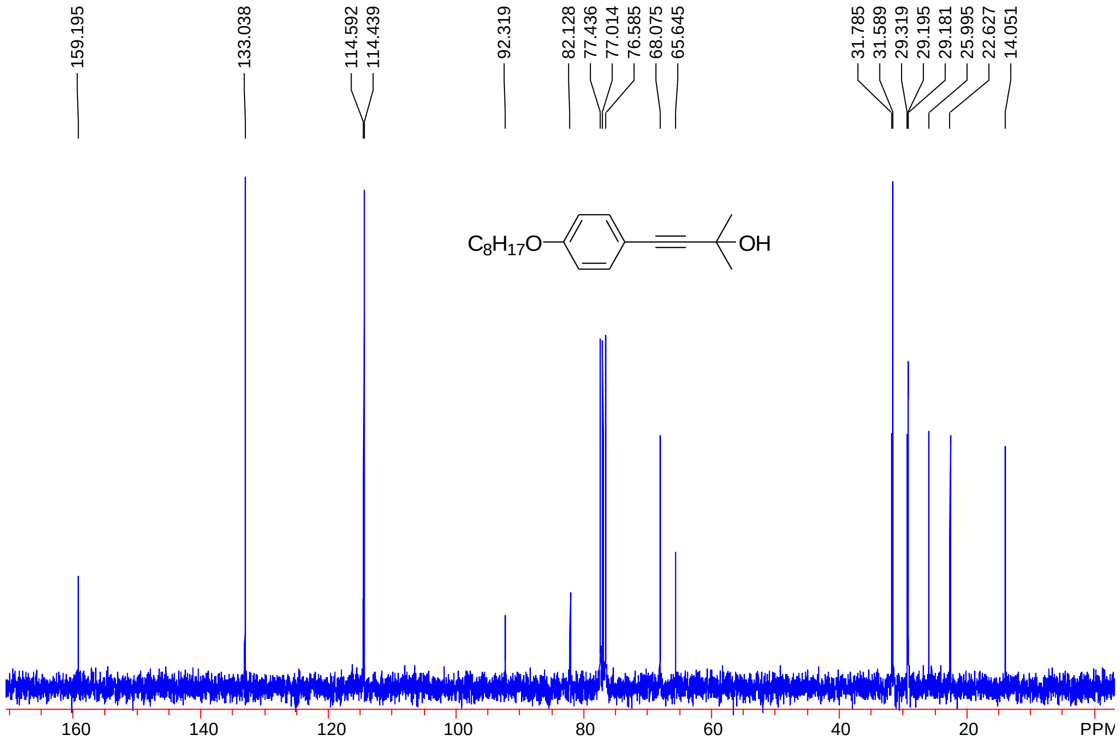

Figure S5: ^{1}H and ^{13}C NMR spectra of **1e**.

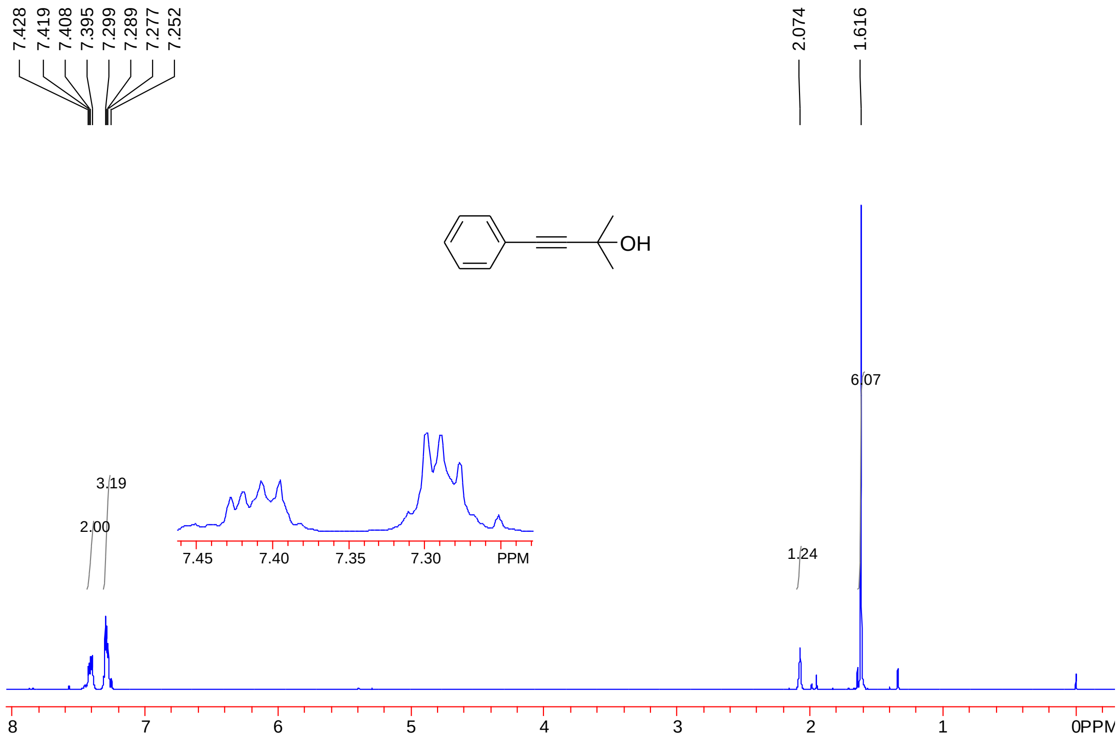

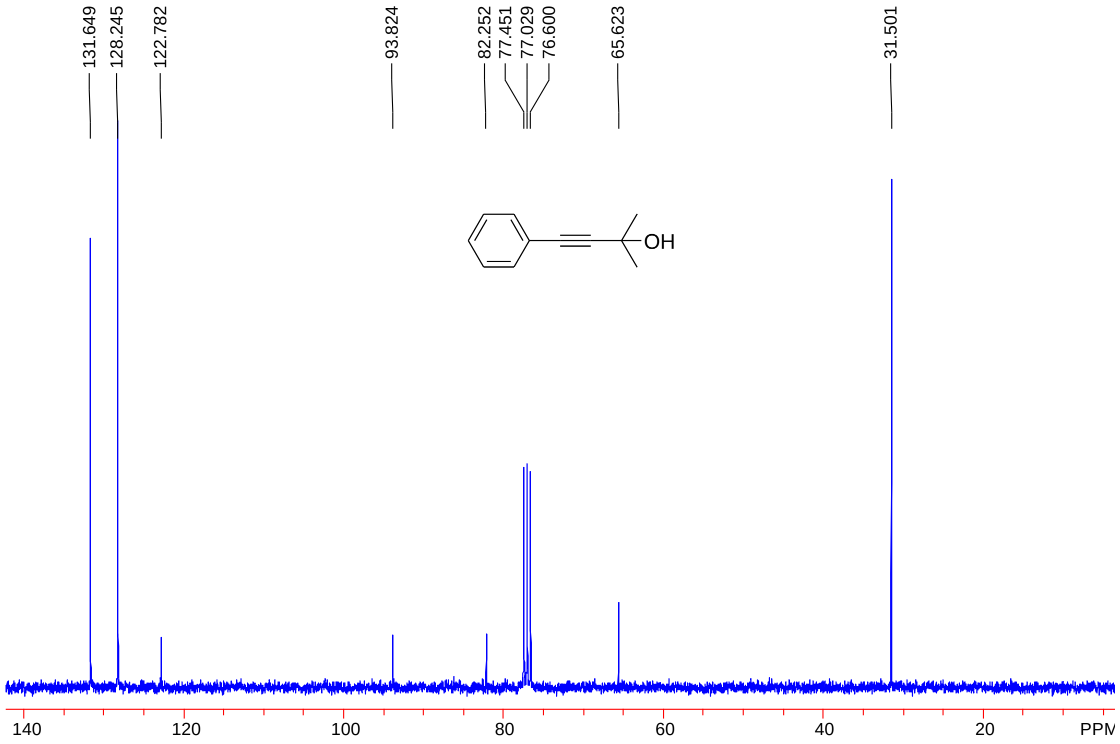

Figure S6: ^{1}H and ^{13}C NMR spectra of **1f**.

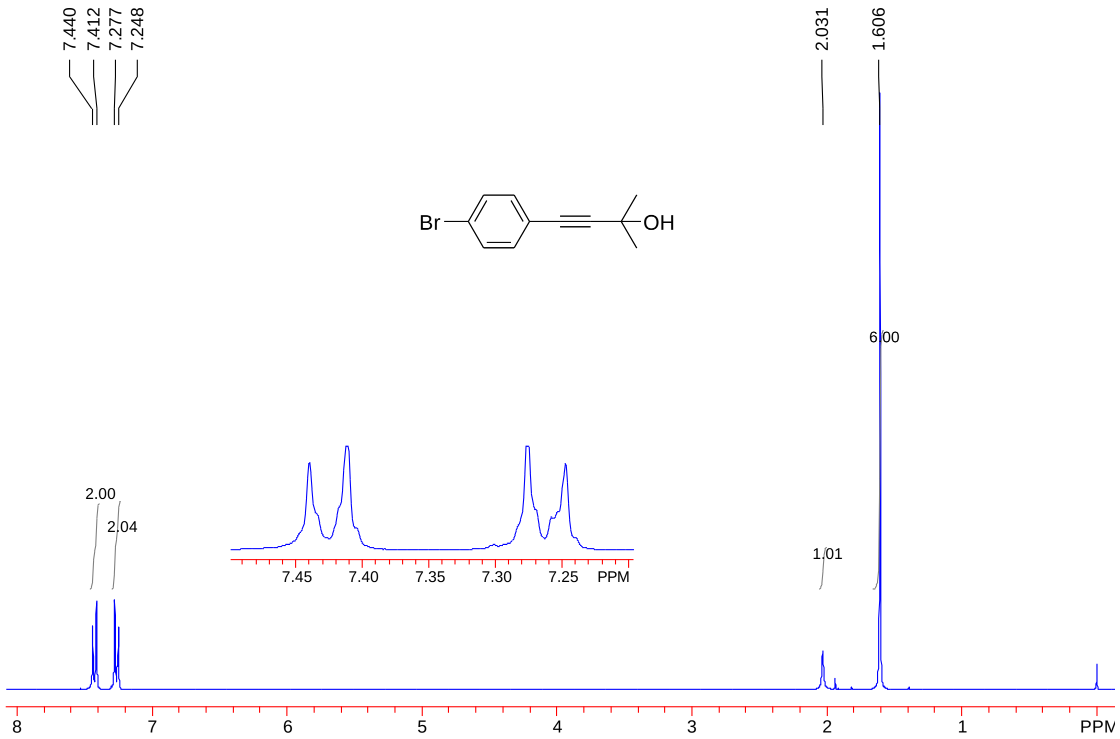

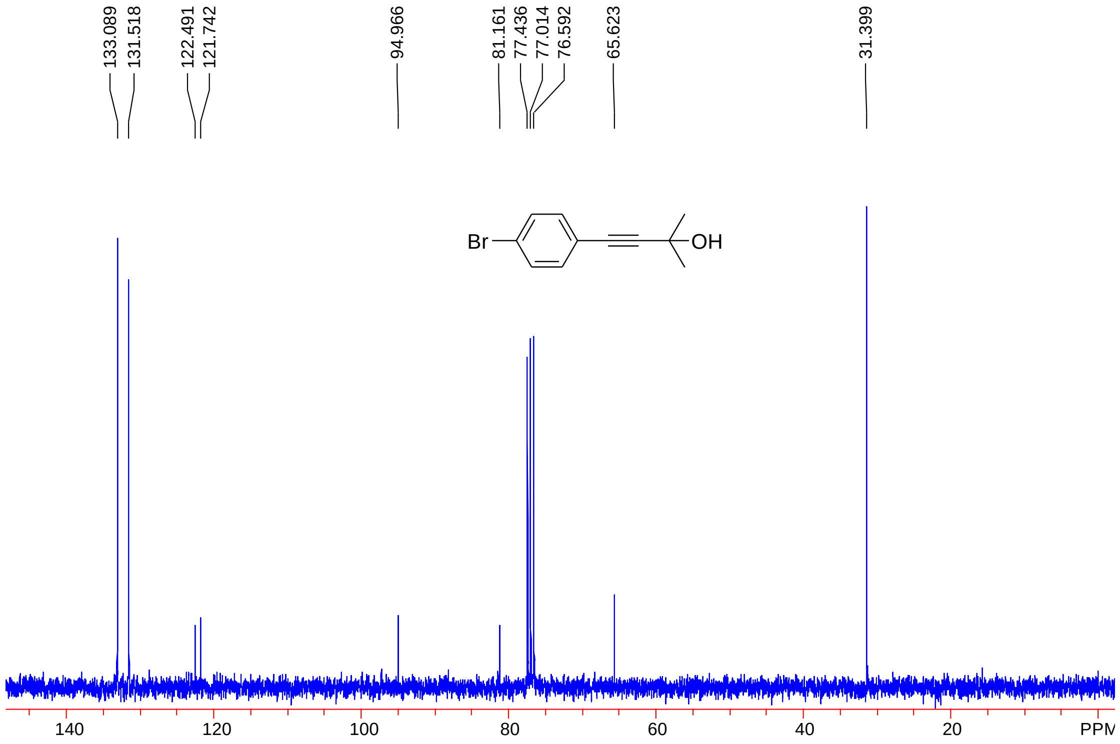

Figure S7: ^{1}H and ^{13}C NMR spectra of **1g**.

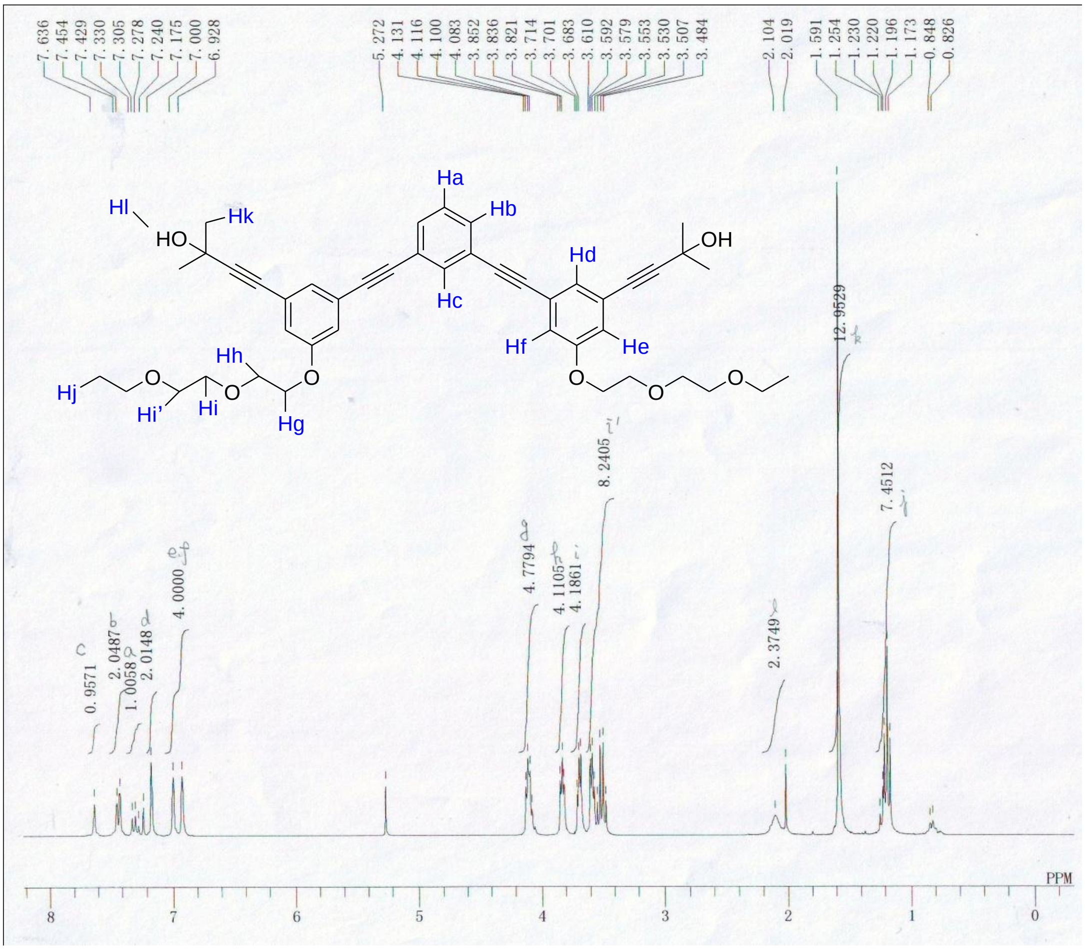

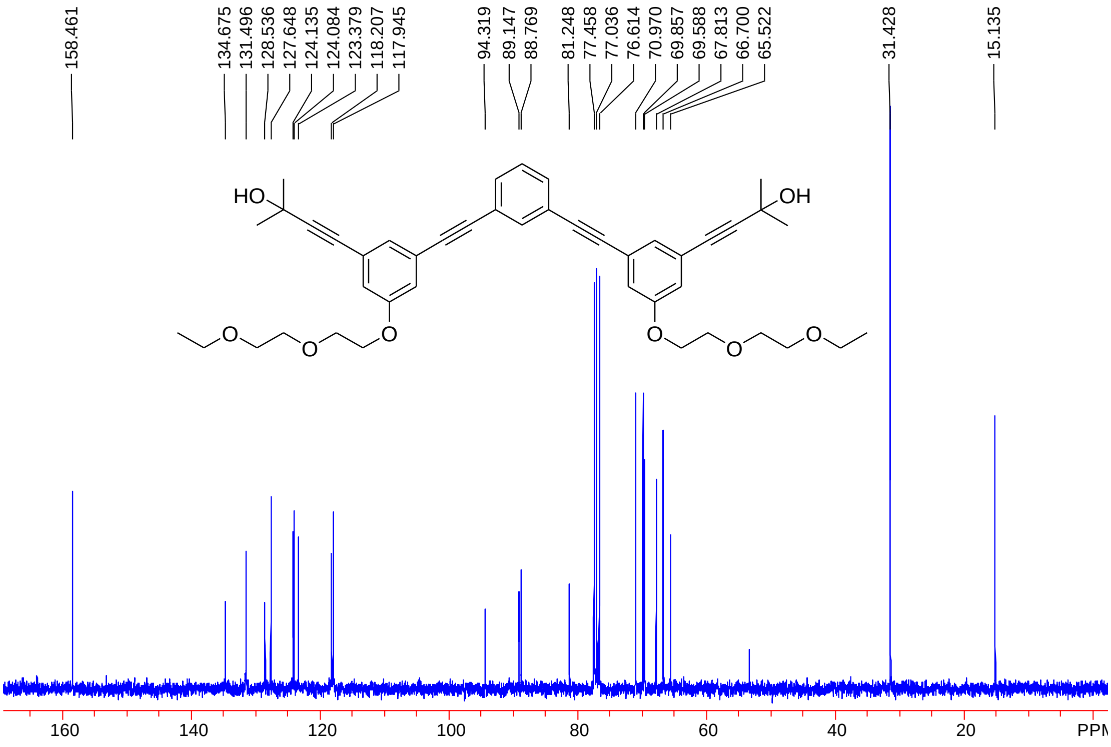

Figure S8: ^{1}H and ^{13}C NMR spectra of **1h**.

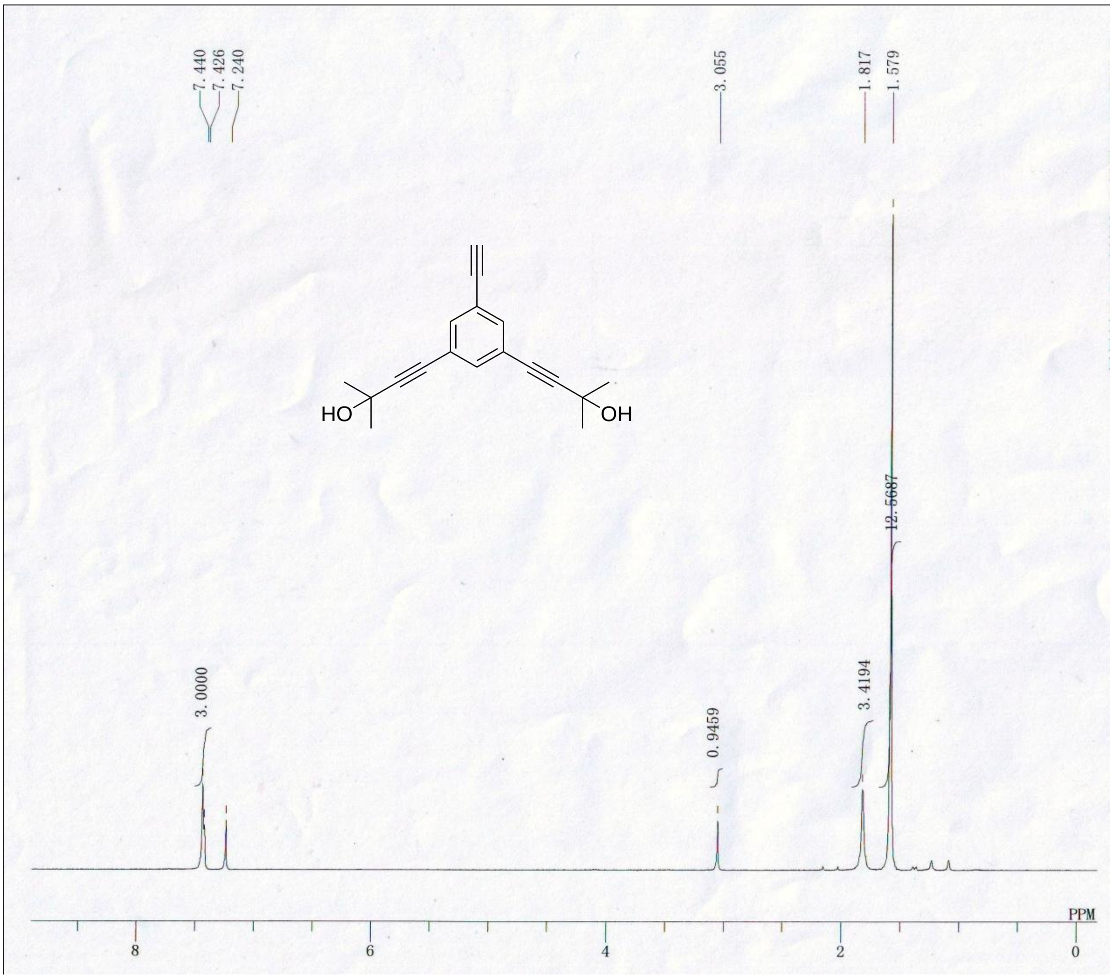

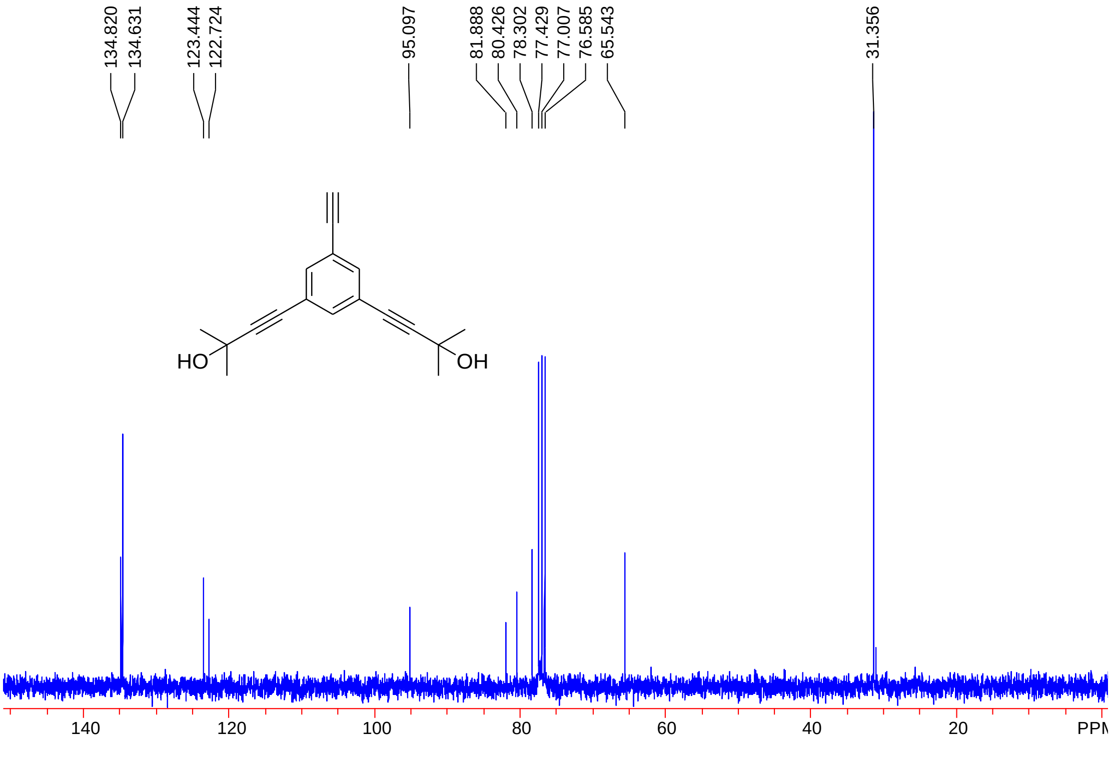

Figure S9: ^{1}H and ^{13}C NMR spectra of **1i**.

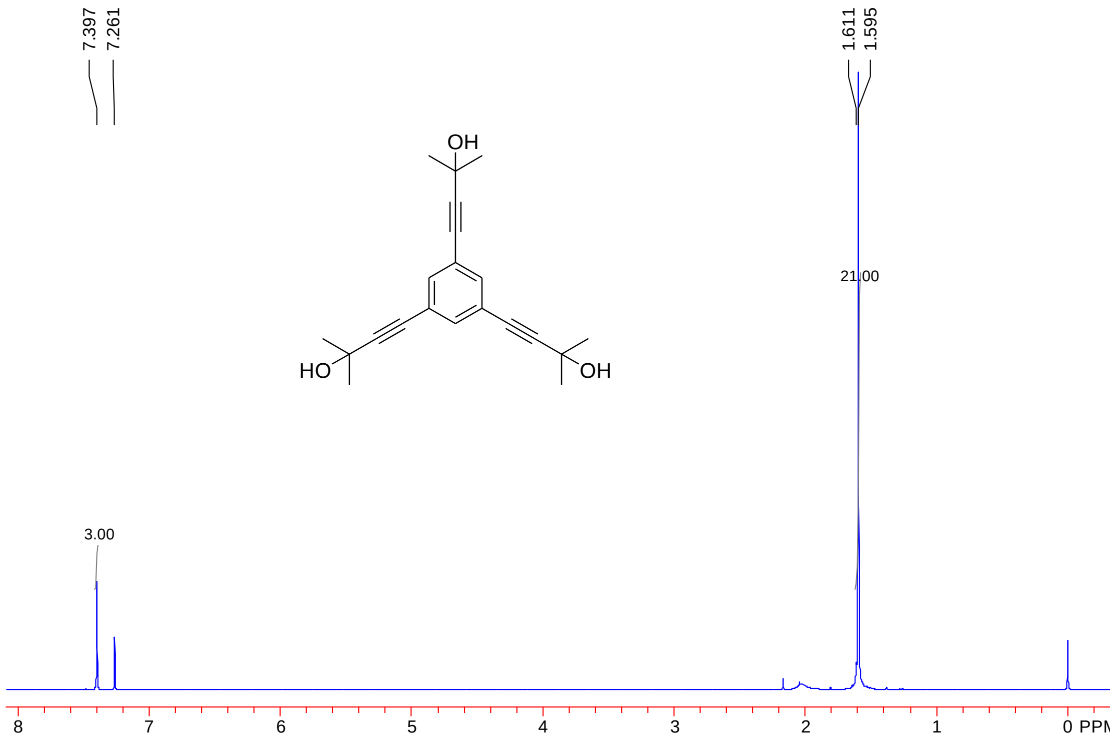

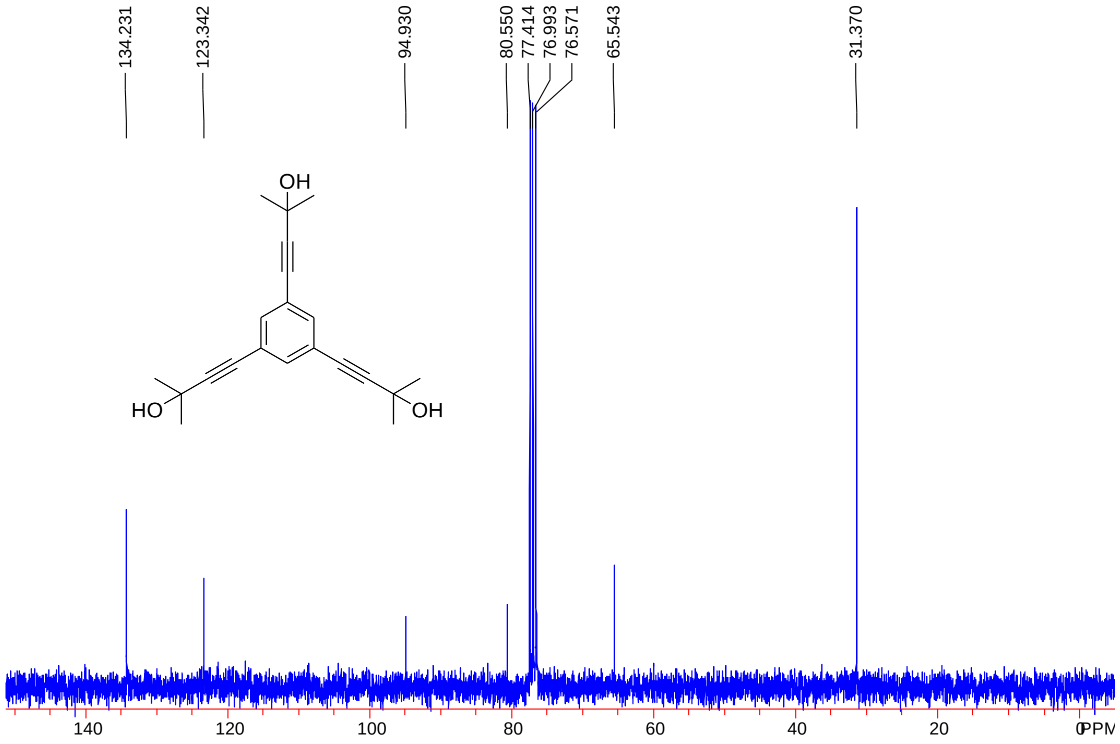

Figure S10: ^{1}H and ^{13}C NMR spectra of **1j**.

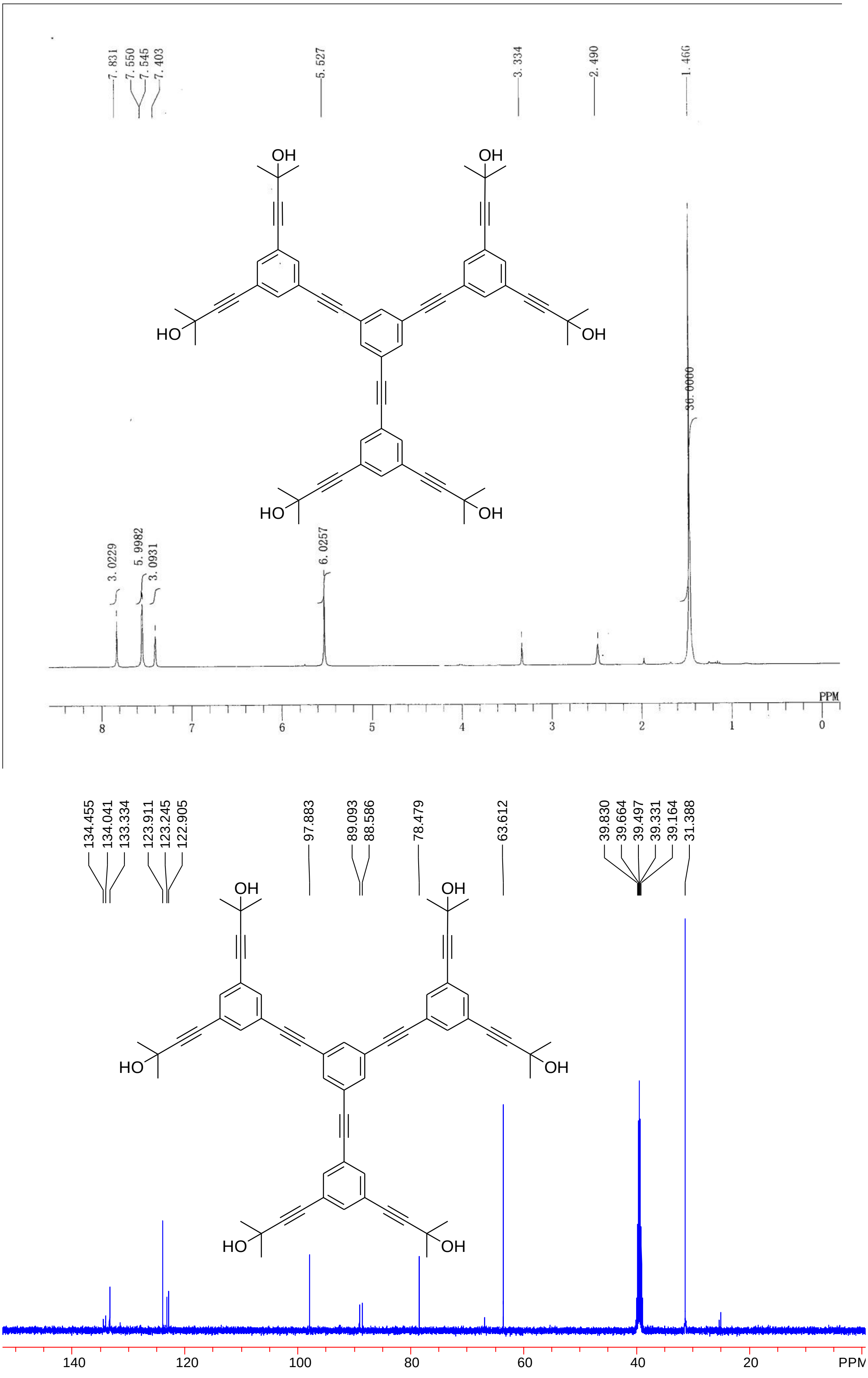

Figure S11: ^{1}H and ^{13}C NMR spectra of **1k**.

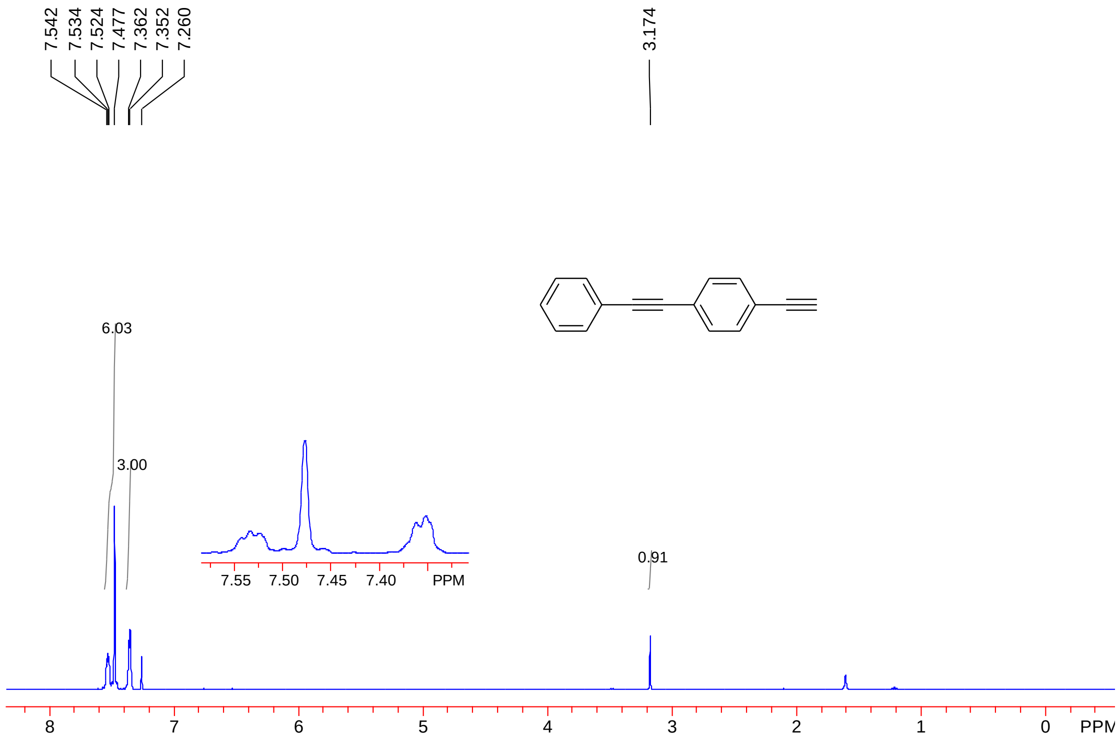

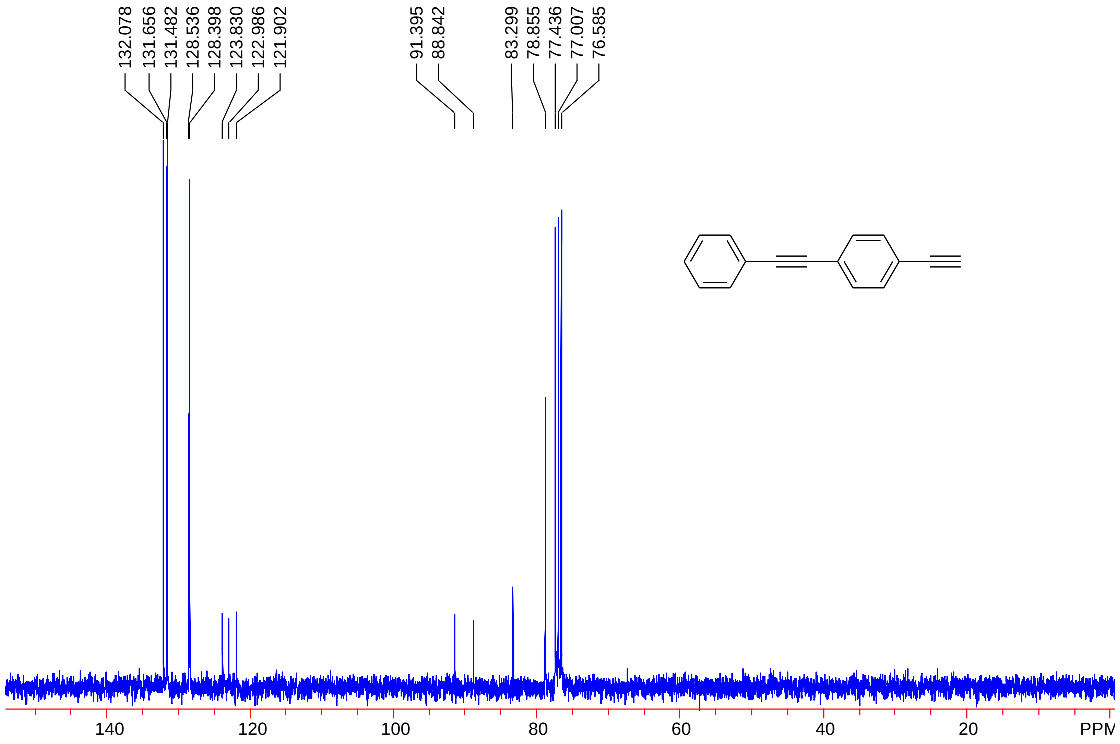

Figure S12: ^{1}H and ^{13}C NMR spectra of **2a**.

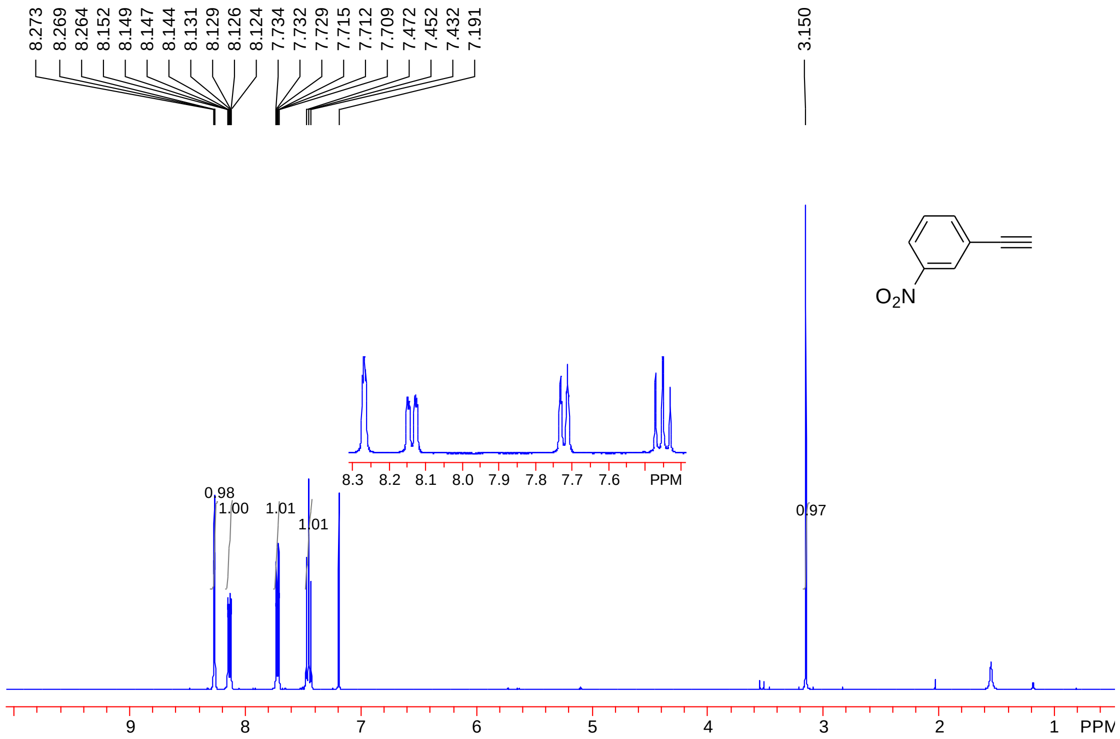

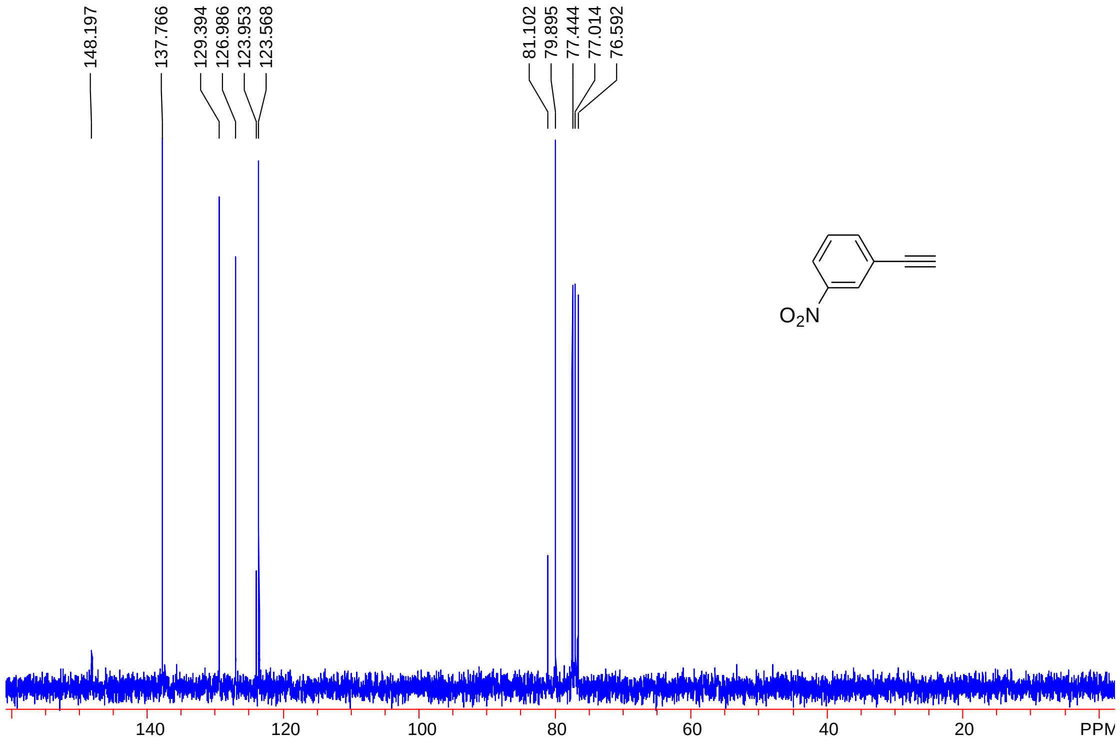

Figure S13: ^{1}H and ^{13}C NMR spectra of **2b**.

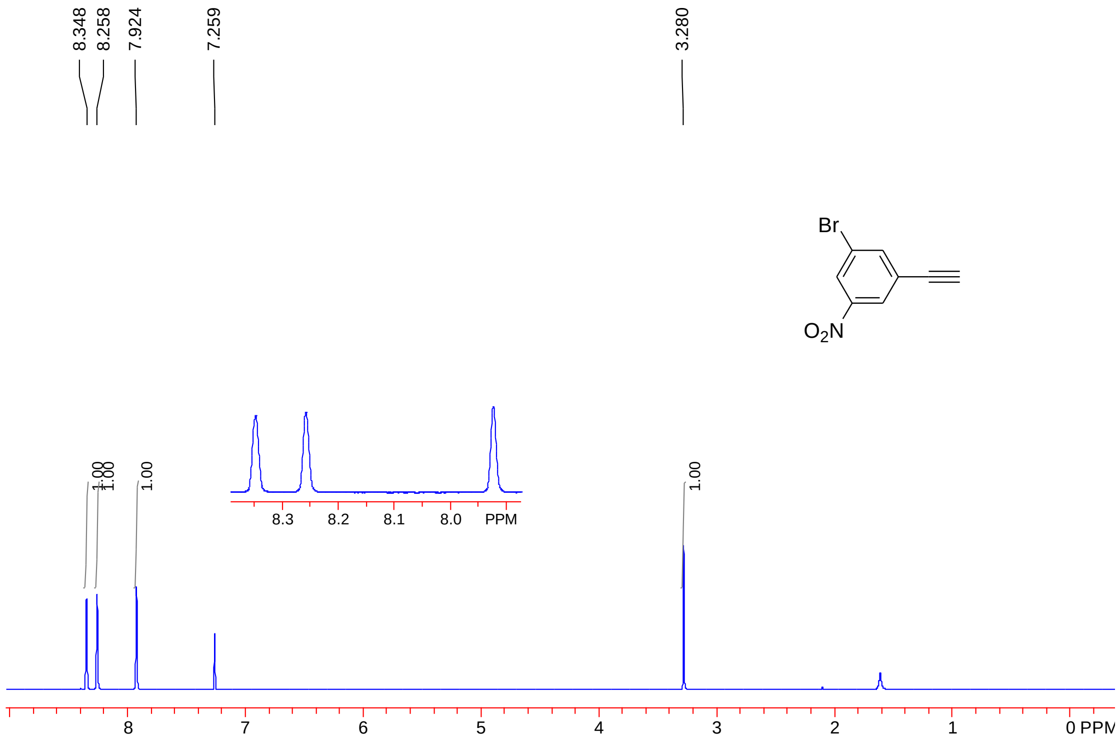

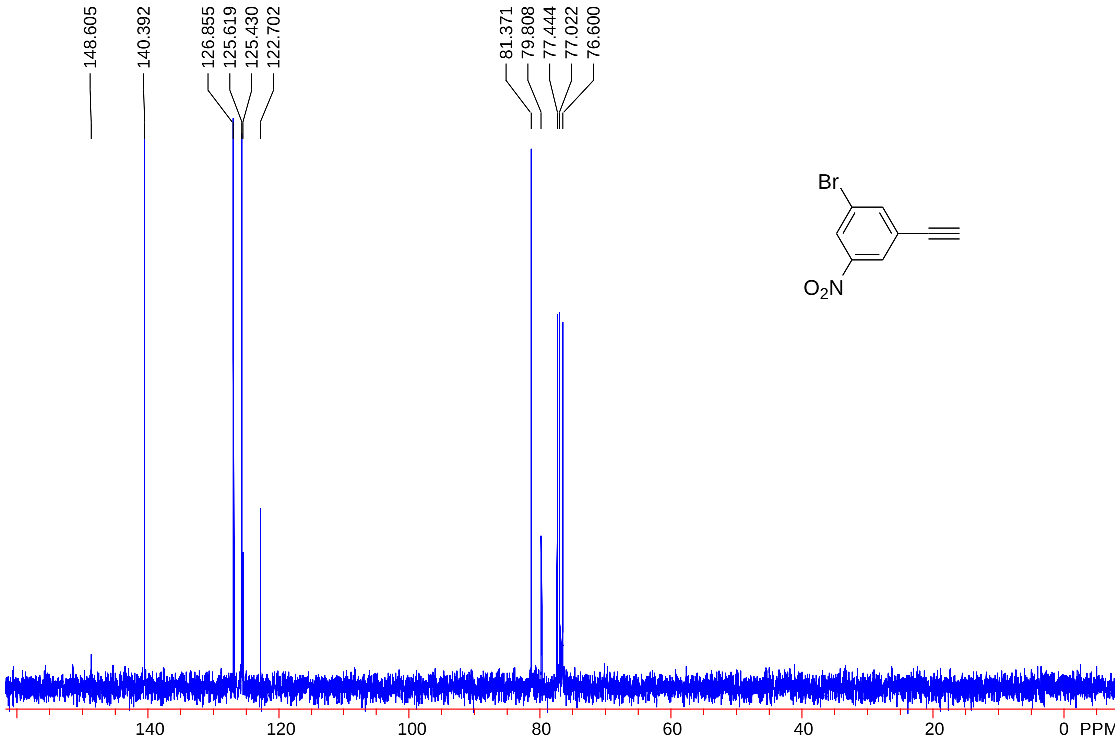

Figure S14: ^{1}H and ^{13}C NMR spectra of **2c**.

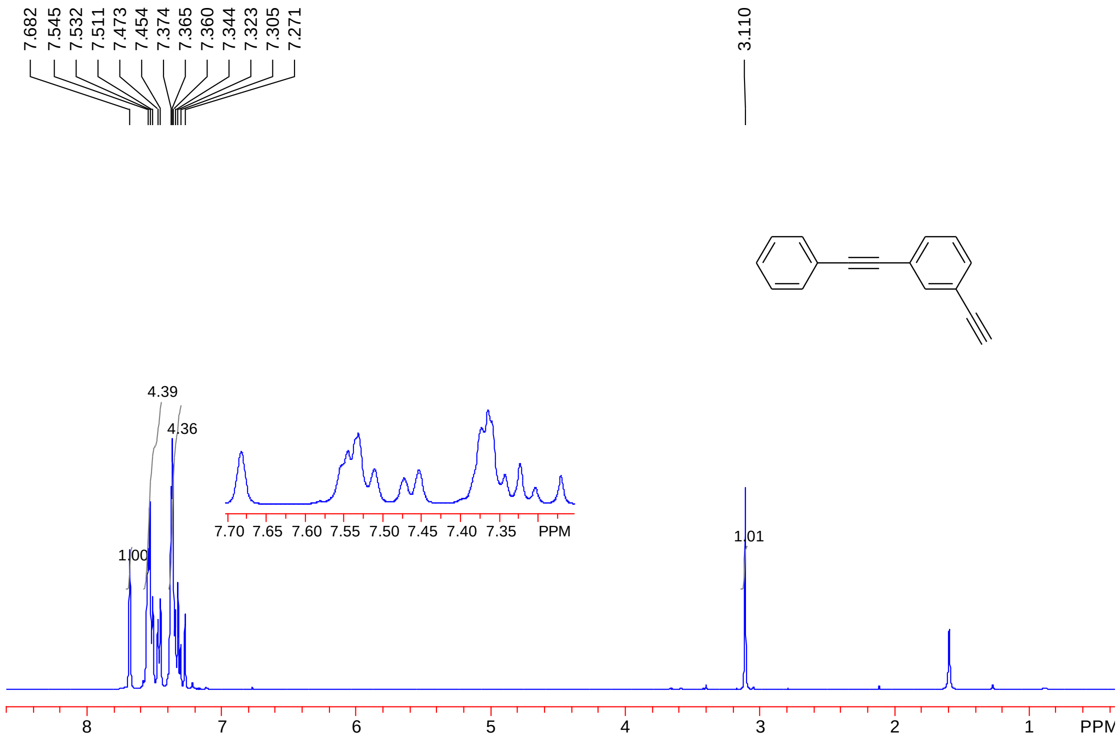

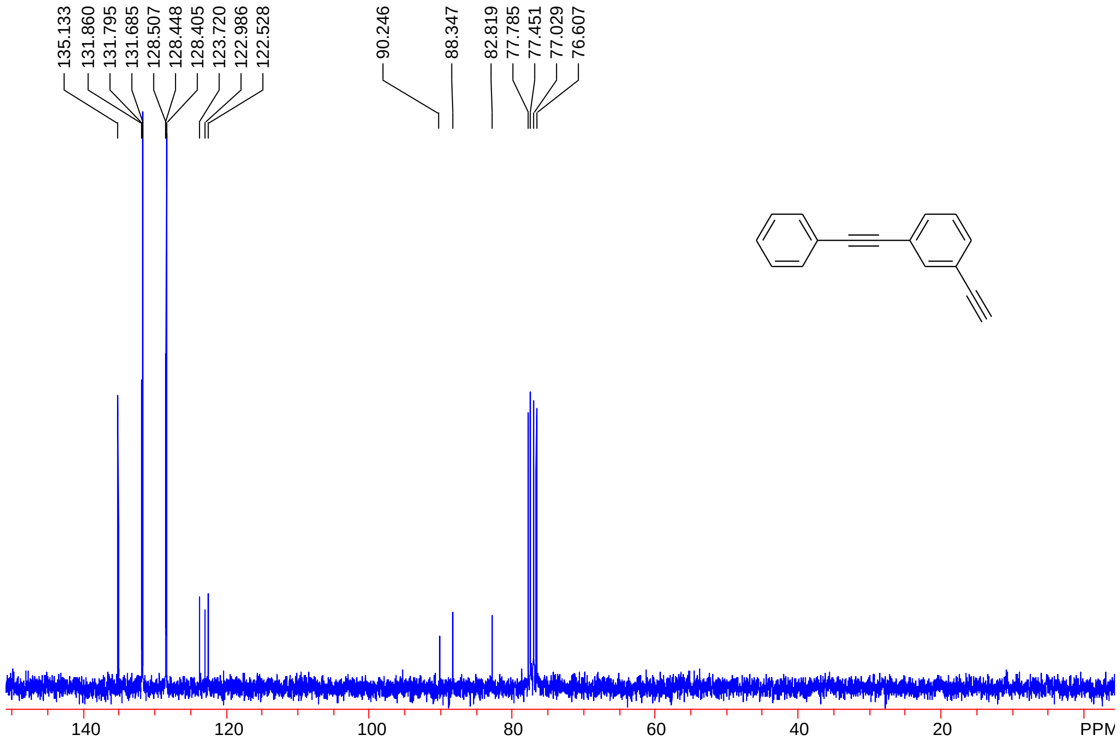

Figure S15: ^{1}H and ^{13}C NMR spectra of **2d**.

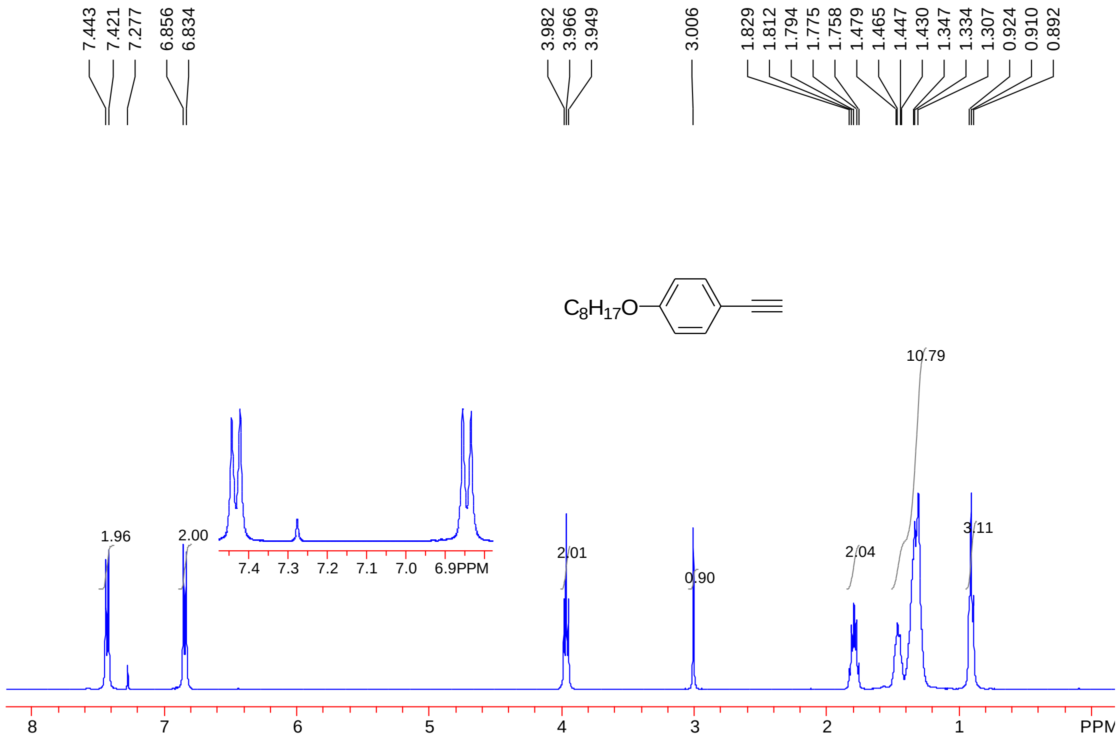

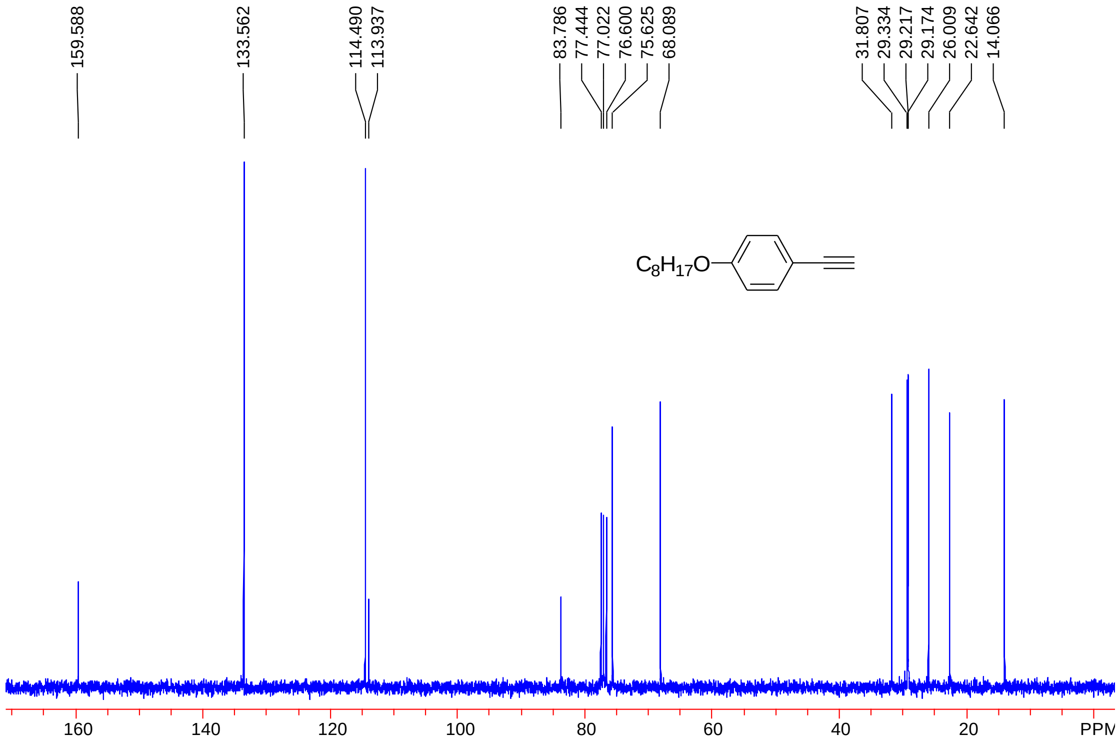

Figure S16: ^{1}H and ^{13}C NMR spectra of **2e**.

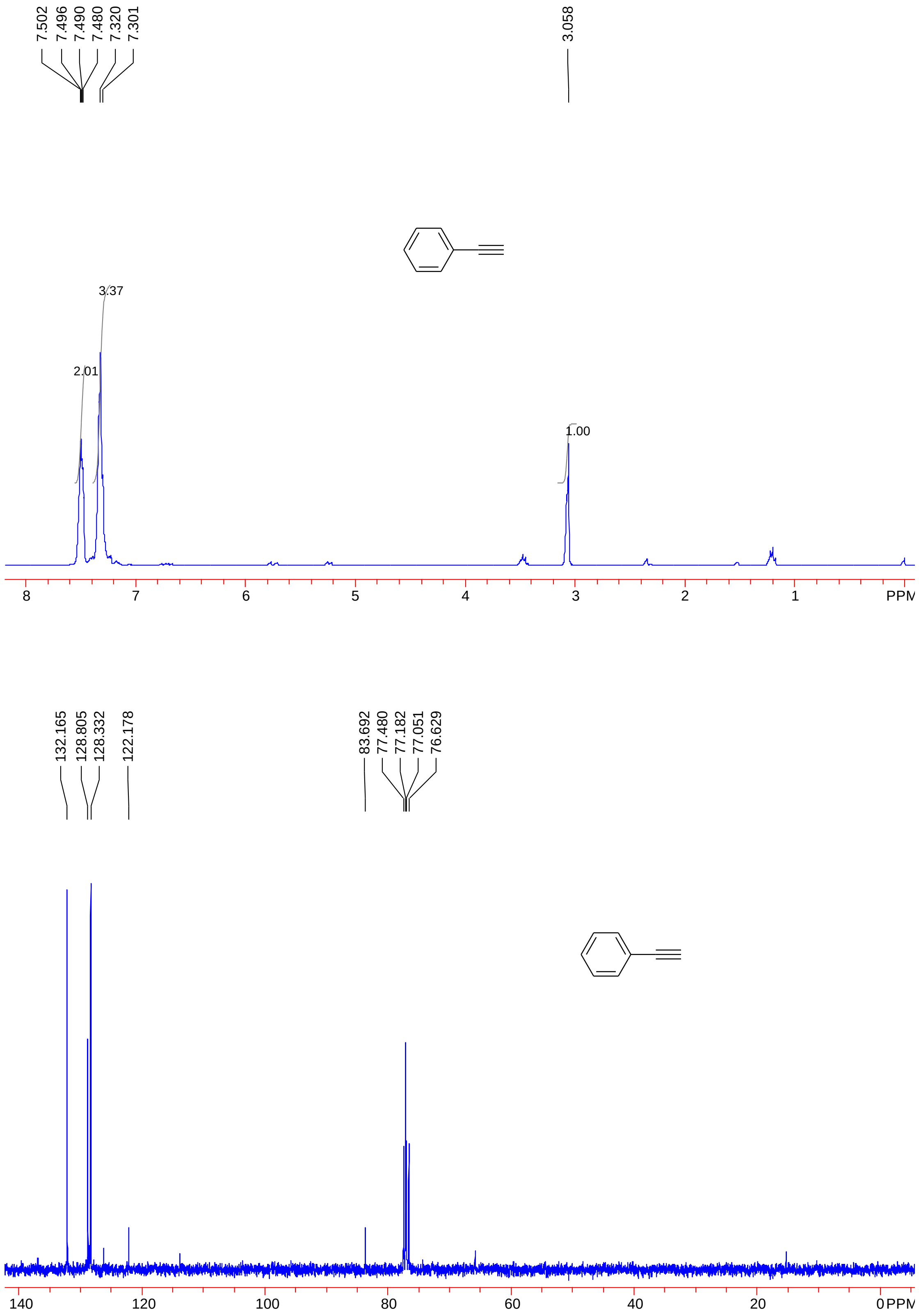

Figure S17: ^{1}H and ^{13}C NMR spectra of **2f**.

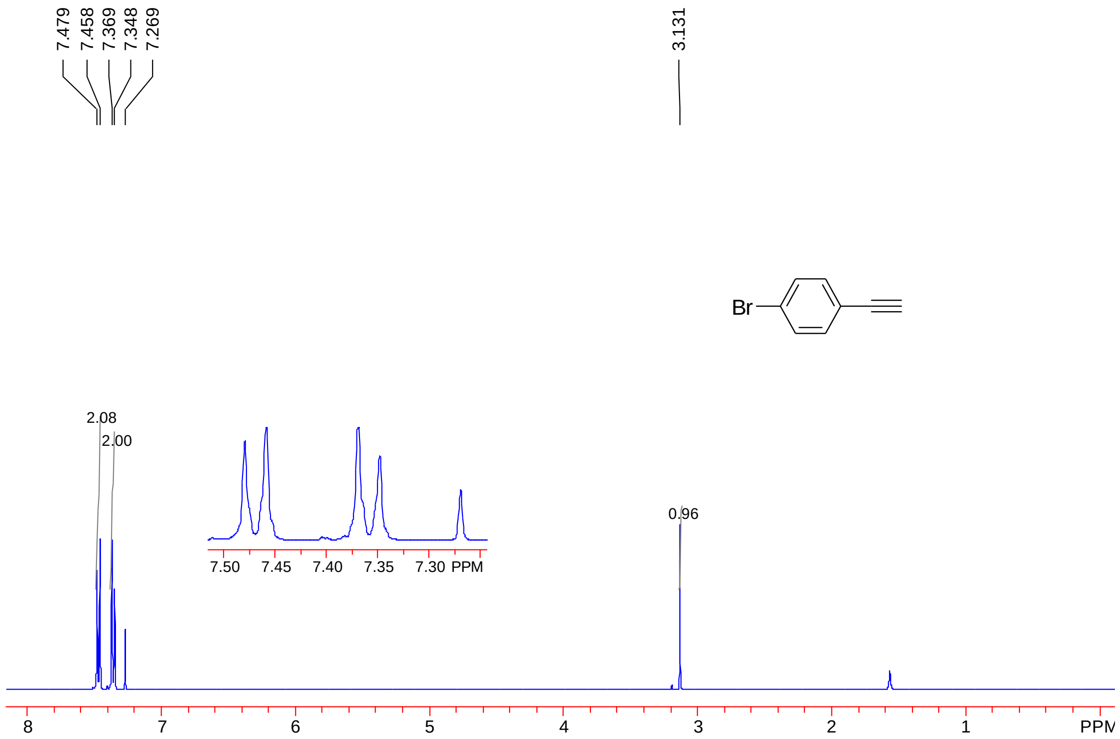

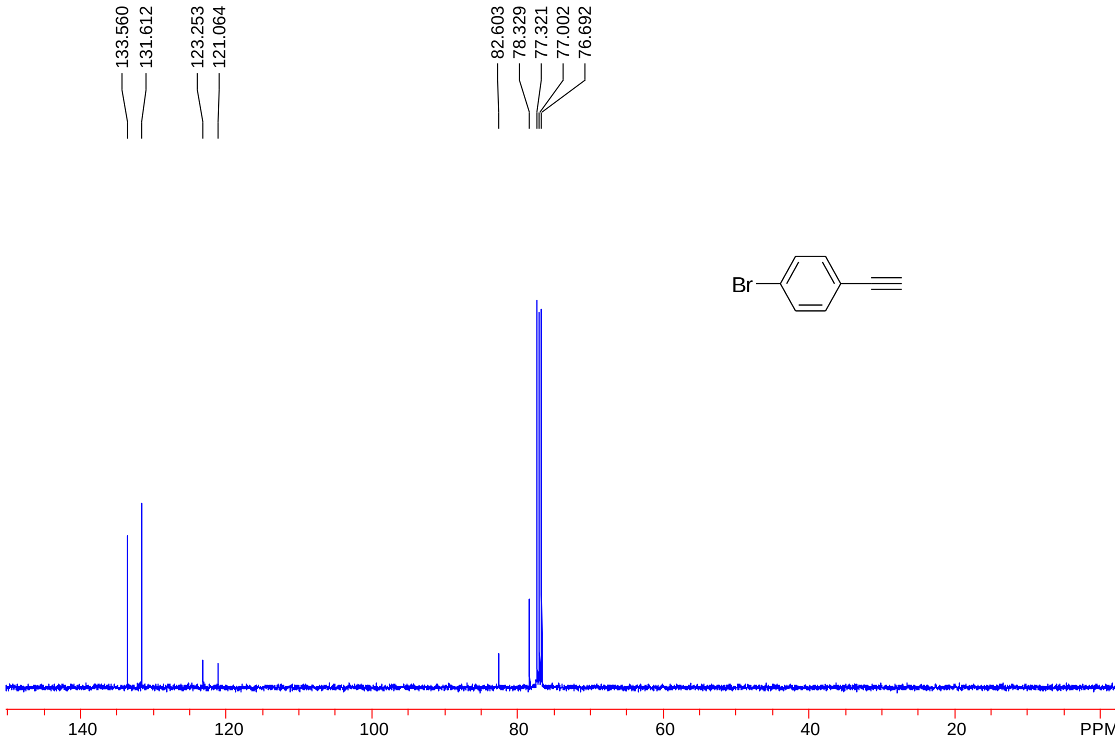

Figure S18: ^{1}H and ^{13}C NMR spectra of **2g**.

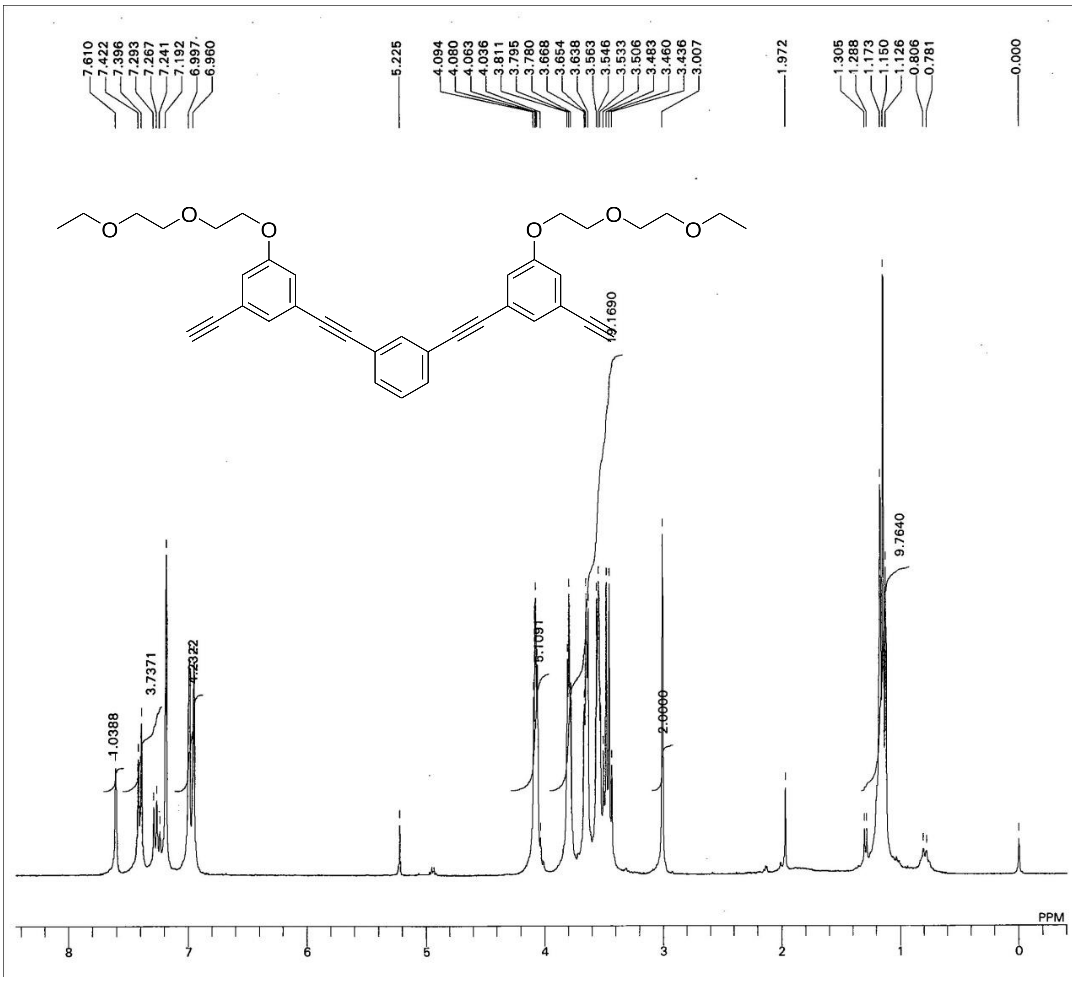

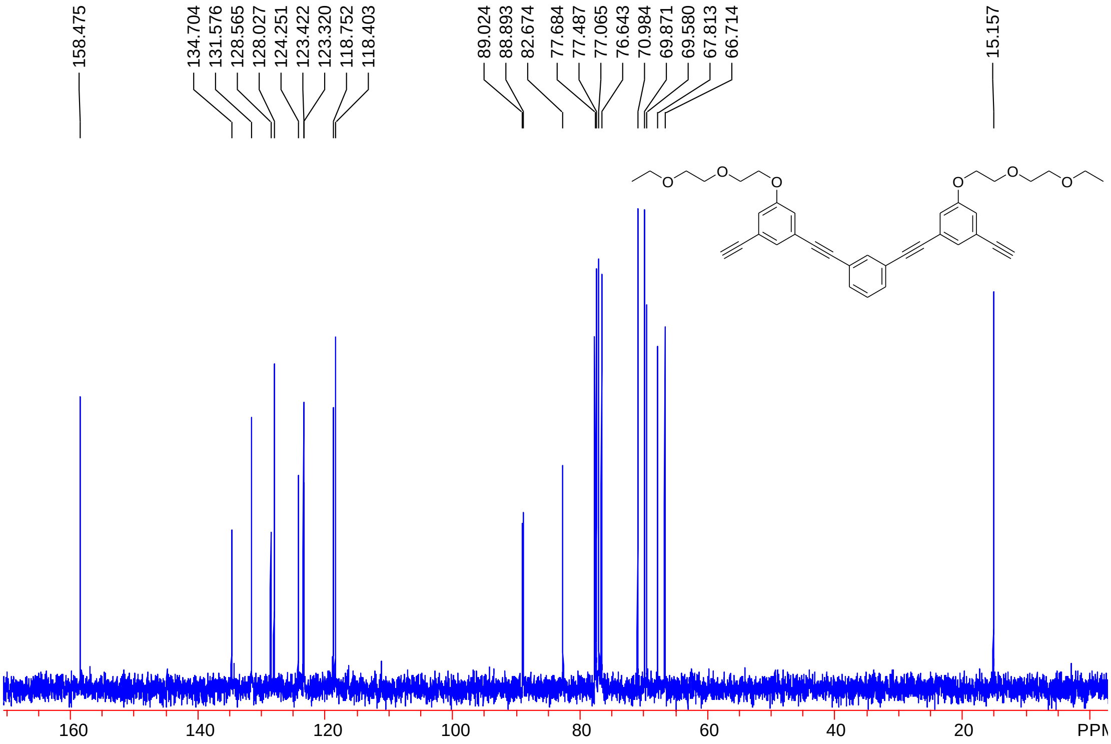

Figure S19: ^{1}H and ^{13}C NMR spectra of **2h**.

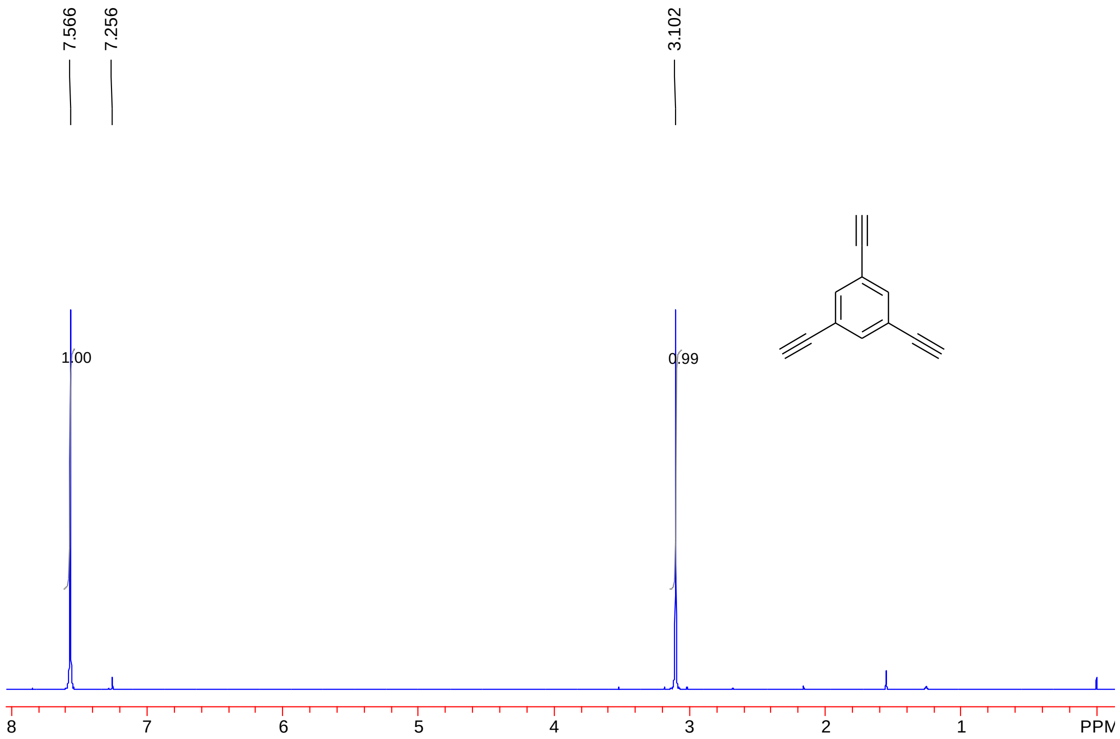

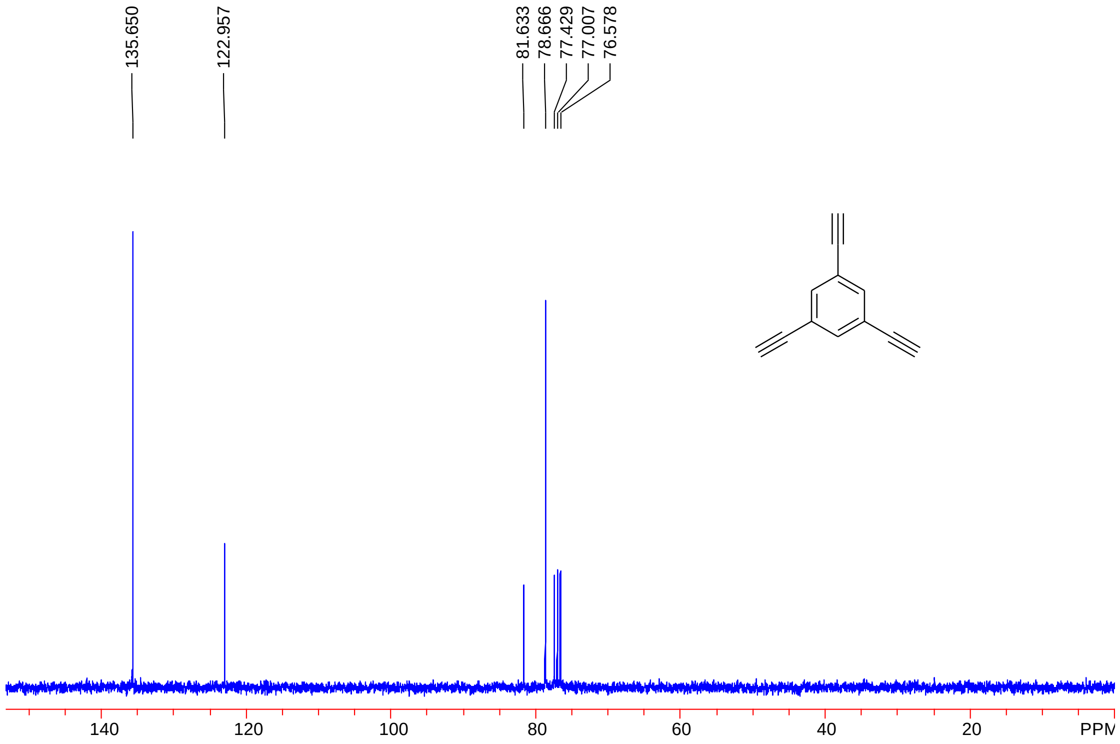

Figure S20: ^{1}H and ^{13}C NMR spectra of **2i**.

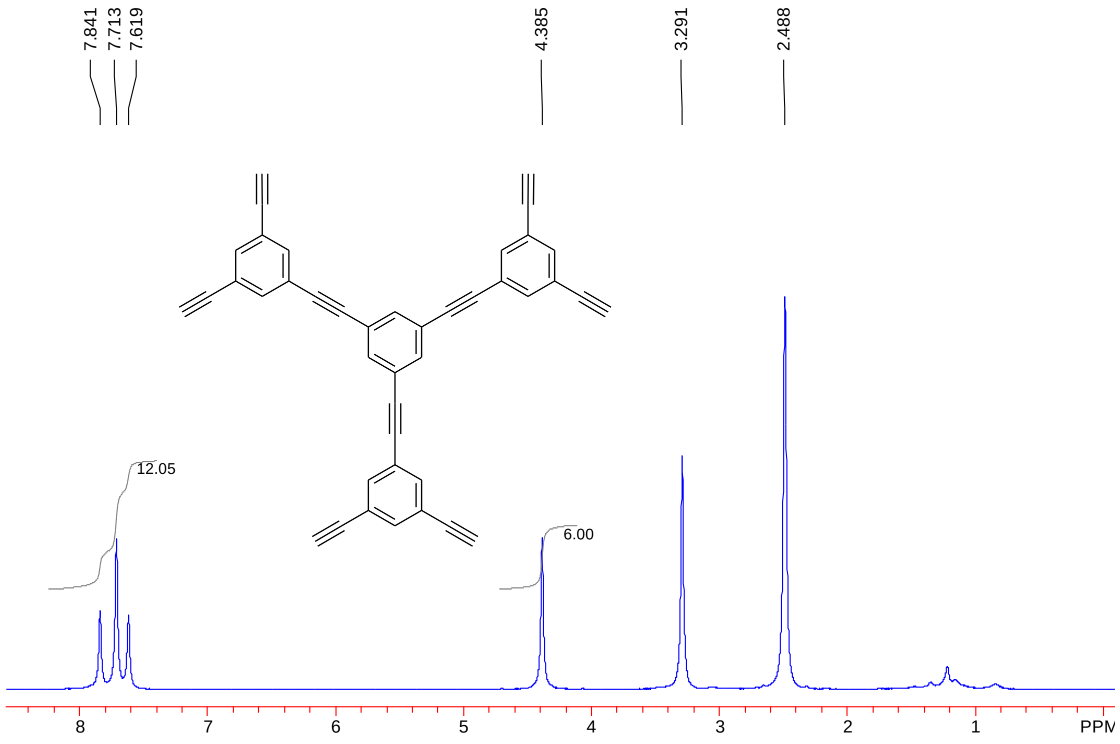

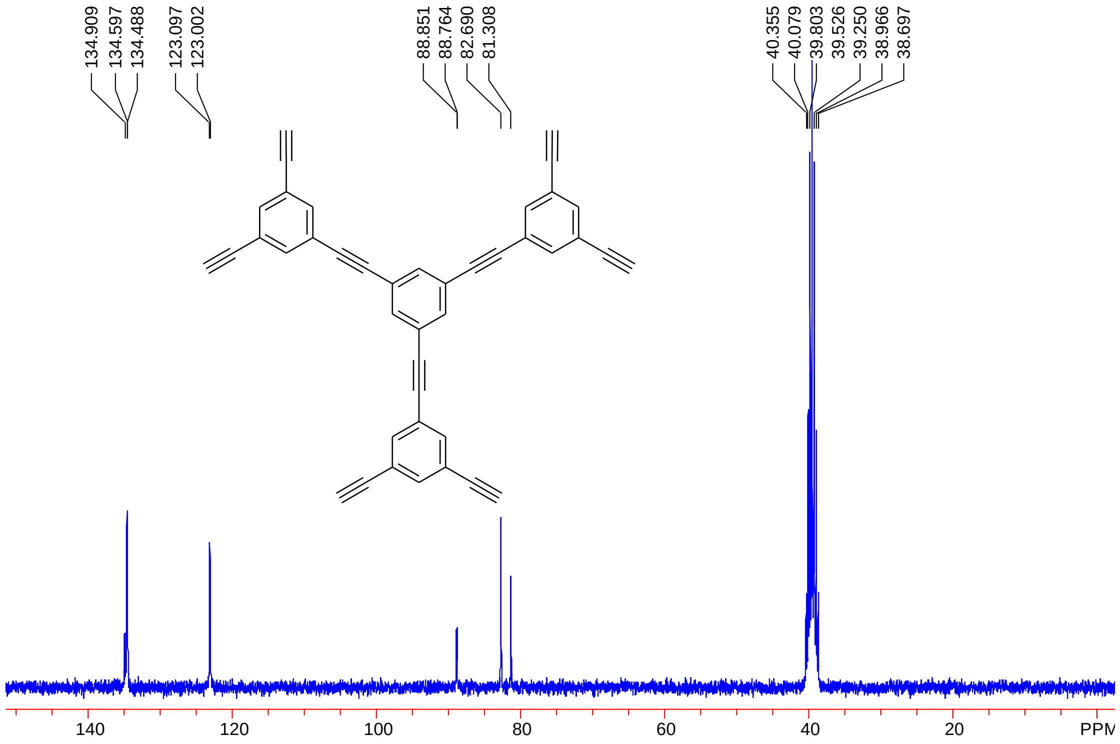

Figure S21: ^{1}H and ^{13}C NMR spectra of **2k**.
